# Supplementary material for: Mixed methods process evaluation of an enhanced community-based rehabilitation intervention for elderly patients with hip fracture
Source: BMJ Open. 2018 Aug 8;8(8):e021486. doi: 10.1136/bmjopen-2018-021486 (PMC6089270; doi:10.1136/bmjopen-2018-021486)
Supplement: Supplementary file 1 [file bmjopen-2018-021486supp001.pdf]

Appendix 1 Case report form

Session number:

Patient Study ID: ☐☐☐☐☐

Date: ____ / ____ / ________ **Total amount of time used for this session:**

Role(s) of person/people conducting visit: Occupational therapist / Physiotherapist / OT assistant / Physiotherapy assistant / Technical instructor / Other

Location of session: Patient's own home / Care home where patient lives permanently / Community hospital outpatients / Acute hospital outpatients / ______________________
Other

1. Please indicate what ***indirect*** care activities you carried out for the patient during this session, and for how long:

**Activity**	**Time (e.g. 10 minutes)**	**Further details**
Referring to another service		
Phone contact with patient		
Contact with MDT about patient		
Patient admin		
Travel to and from session		
Other, please specify.		

2. Please indicate what ***direct*** care activities you carried out for the patient during this session, and for how long:

**Activity**	**Time (e.g. 20 minutes)**	**Further details**
Assessment/re-assessment		
Physical exercises		
Activities of daily living practice		
Workbook		
Discussion of care and progress		

**Activity**	**Time (e.g. 20 minutes)**	**Further details**
Discussion of referral to a follow on service e.g. social groups, falls groups, outpatients physio		
Travel to and from session		
Other, please specify		

3. If you spent time on the ***workbook*** in the session, please indicate what this involved:

**Activity**	**Time (e.g. 5 minutes)**	**Further details**
Goal setting		
Reviewing progress		
Answering patient questions stimulated by workbook		
Emotional support		
Other, please specify		

Appendix 2 Focus group topic guides

Patient and carer topic guide – control group

- What was your experience of the rehabilitation you received after your hip fracture, specifically regarding physiotherapy and exercise?
- What went well, what could be improved? Having experienced fracture and the rehabilitation involved, what would you recommend to improve care?
- If there are any attending the focus group, what were the experiences of participants who were discharged to nursing/residential care, or those who were more dependent on carers?

- Did your therapists give you exercises to do?
    - How confident did you feel about practising them?
    - Did your confidence change over time? What changed this?
- Were you given any written information to keep with you?
    - How helpful was this? Was there any other information you would have found useful?
- How involved did you feel in planning your recovery and rehabilitation?
- Often people say they were afraid of falling again after having a hip fracture, did you experience this?
    - Was there anything in your rehabilitation that helped reduce the fear of falling?
    - Did you attend a falls prevention class? How did you find out about this and was it useful?
- How did you feel about being approached to take part in the study?
    - Did you understand the information you were given?

    - What made you decide to take part?
    - How did you feel about the questions you were asked in the hospital and at the follow up, especially about how long they took and how well you were able to understand the questions and their relevance?
- How did you feel about not knowing which study group you would be in when you agreed to take part?
    - How would you have felt if you had been in the other group?
- Did you do some exercise tests with your community physiotherapists at the end of the study?
    - How did you feel about being asked to do these?
- Has anything changed for you as a consequence of being in the study?
    - If you were asked to take part now, would you make the same decision?

Patient and carer topic guide – intervention group

- What was your experience of the rehabilitation you received after your hip fracture?
    - What went well, what could be improved?
    - If there are any attending the focus group, what were the experiences of participants who were discharged to nursing/residential care, or those who were more dependent on carers?
- TASK - Rank the following aspects of the intervention according to how useful they were:
    - Extra time with a therapist
    - Information in the workbook
    - Being involved in setting your own goals

    - Keeping a diary to look at progress
- Discussion of ranking
- Did you feel confident to suggest goals to your therapists and how did that help you?
- What did you like best about the workbook?
    - Was there anything you felt the workbook was missing?
- Were you aware of the extra sessions which you received because you were in the study?
    - Were these sessions used differently to the care as usual sessions?
    - Did you notice any improvements in your recovery as a result of this extra time?
- Did your therapists give you exercises to do?
    - How confident did you feel about practising them?
    - Did your confidence change over time? What changed this?
- Often people say they were afraid of falling again after having a hip fracture, did you experience this?
    - Was there anything in your rehabilitation that helped reduce the fear of falling?
    - Did you attend a falls prevention class? How did you find out about this and was it useful?
- How did you feel about being approached to take part in the study?
    - Did you understand the information you were given?
    - What made you decide to take part?
    - How did you feel about the questions you were asked in the hospital and at the follow up, especially about how long they took and how well you were able to understand the questions and their relevance?

- Did you do some exercise tests with your community physiotherapists at the end of the study?
    - How did you feel about being asked to do these?
- How did you feel about not knowing which study group you would be in when you agreed to take part?
    - How would you have felt if you had been in the other group?
- Has anything changed for you as a consequence of being in the study?
    - If you were asked to take part now, would you make the same decision?

Healthcare professional topic guide

- Experiences of the enhanced rehabilitation intervention
    - What went well?
    - What could be improved?
- Experiences of extra therapist time
- Awareness of extra time available to patients in the study
    - How did if work, what went well what needs to be improved
    - How did you use the extra time with patients? Do you think it made a difference?
- Views of the Workbook
    - Did you use it?
    - If so how, what was useful, what could be improved, was anything missing you think would have been helpful?

- Do you feel the intervention made any difference to the way patients engaged with you and participated in their rehabilitation?
    - If so how/if not why?
    - What was your experience the goal setting and feedback in the work book? Similar/different to usual way you work/good points/areas for improvement
    - What was your experience of the information sections?
      Useful to you/useful to patients/anything you weren't aware of before/ anything that was missing?
- Experiences of working with patients with cognitive impairments and their carers
    - How did the intervention go?

- Experiences of working with patients who were discharged to long term residential/nursing care
    - How did the intervention go?
- Feasibility of physiotherapists conducting the physical measures at 3 month follow-up
- Acceptability of the outcome measures for patients
    - Timing; Number; Content
- Anything else we haven't talked about you think has been important in working with this intervention?

Appendix 3 Rehabilitation pathways in usual care on discharge from the central acute hospital following surgical repair of hip fracture

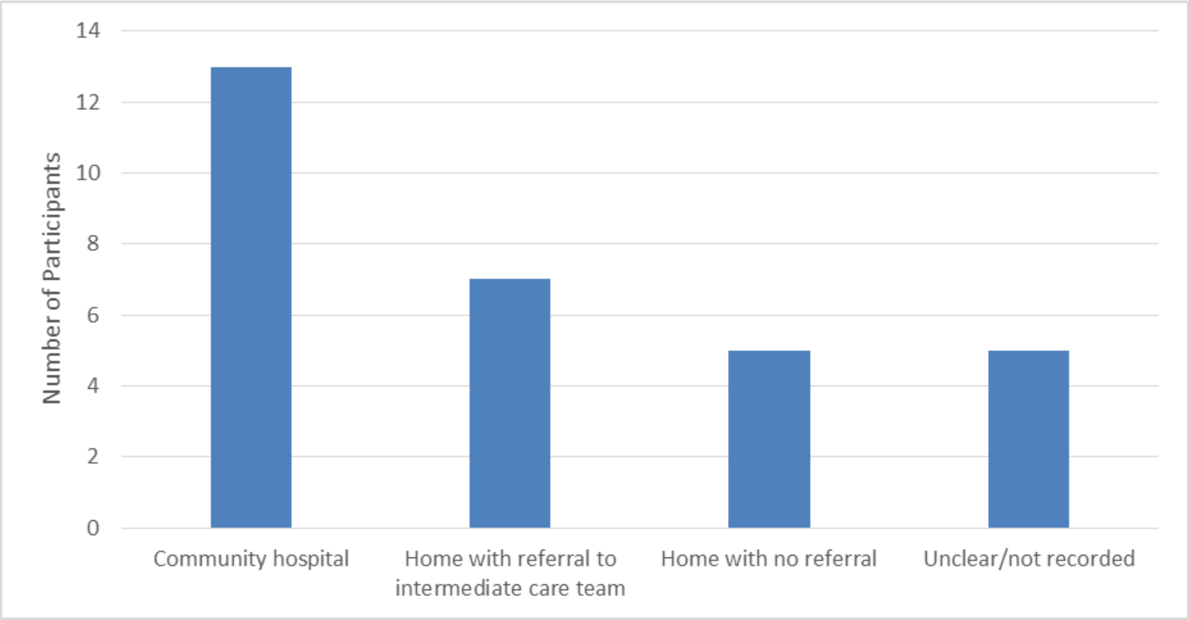

Appendix 4 Percentage of home visits conducted by different members of the rehabilitation team for usual care

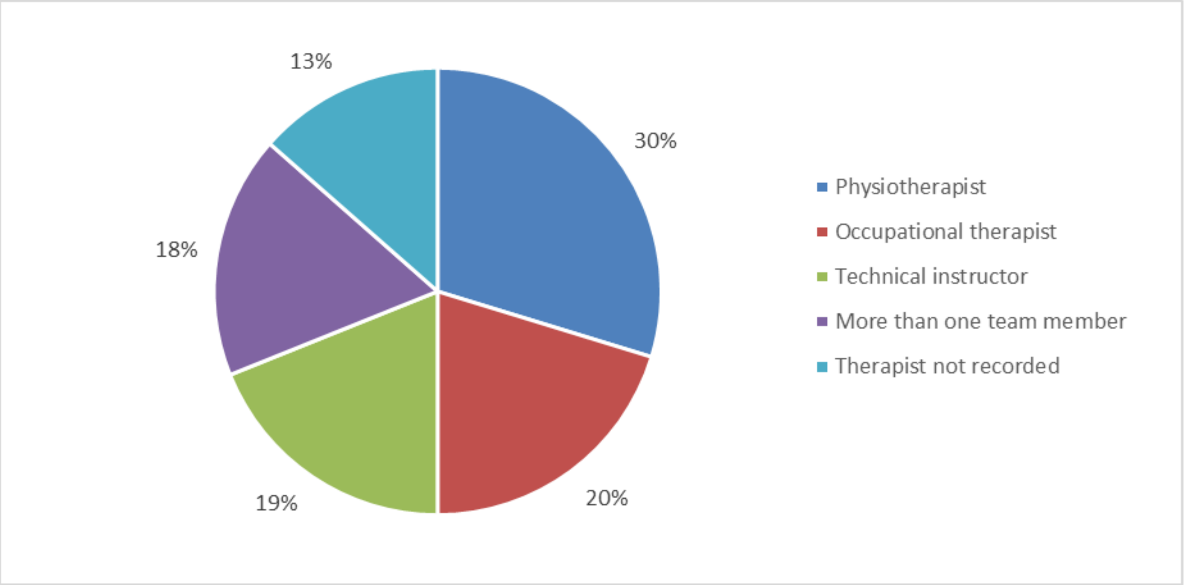

Appendix 5 Reasons for missing data on intervention session use

Reason	No. of participants	Area
Withdrawn	4	Gwynedd, Anglesey, Conwy and Denbighshire
Area not covered by therapist	1	Gwynedd and Anglesey
Declined intervention sessions	1	Conwy and Denbighshire
Could not be contacted to arrange sessions	1	Conwy and Denbighshire
Received intervention but therapist not returned paperwork	1	Gwynedd and Anglesey
Therapist delivering sessions moved to different area and could not complete intervention	1	Gwynedd and Anglesey

Appendix 6 Correlation analysis of Barthel Index with measures of self-efficacy

Self-Efficacy Measures	Barthel Index at baseline<br>Correlation (95% Confidence Intervals)	Barthel Index at 3 months<br>Correlation (95% Confidence Intervals)
General Self-Efficacy Scale (Baseline and 3 Months)	r_{55}= 0.43 (0.12 to 0.68), p=0.001	r_{37}= 0.59 (0.23 to 0.82), p<0.001
Falls Efficacy Scale – International* (3 Months)	Not Collected	r_{34}= -0.68 (-0.83 to -0.45), p<0.001
Self-Efficacy for Exercise Scale (3 Months)	Not Collected	r_{33}= 0.62 (0.26 to 0.82), P<0.001

* High values represent high levels of fear of falling
