# Supplementary material for: Mixed methods process evaluation of an enhanced community-based rehabilitation intervention for elderly patients with hip fracture
Source: BMJ Open. 2018 Aug 8;8(8):e021486. doi: 10.1136/bmjopen-2018-021486 (PMC6089270; doi:10.1136/bmjopen-2018-021486)
Supplement: Author's manuscript [file bmjopen-2018-021486.draft_revisions.pdf]

BMJ Open

BMJ Open is committed to open peer review. As part of this commitment we make the peer review history of every article we publish publicly available.

When an article is published we post the peer reviewers' comments and the authors' responses online. We also post the versions of the paper that were used during peer review. These are the versions that the peer review comments apply to.

The versions of the paper that follow are the versions that were submitted during the peer review process. They are not the versions of record or the final published versions. They should not be cited or distributed as the published version of this manuscript.

BMJ Open is an open access journal and the full, final, typeset and author-corrected version of record of the manuscript is available on our site with no access controls, subscription charges or pay-per-view fees (http://bmjopen.bmj.com).

If you have any questions on BMJ Open's open peer review process please email info.bmjopen@bmj.com

BMJ Open

Mixed methods process evaluation of an enhanced rehabilitation intervention for elderly hip fracture patients.

Journal:	*BMJ Open*
Manuscript ID	bmjopen-2018-021486
Article Type:	Research
Date Submitted by the Author:	24-Jan-2018

Complete List of Authors:	Roberts, Jessica; Bangor University, North Wales Centre for Primary Care Research<br>Pritchard, Aaron; Betsi Cadwaladr University Health Board, Research and Development<br>Williams, Michelle; Bangor University<br>Totton, Nikki; Bangor University College of Health and Behavioural Sciences, North Wales Organisation for Randomised Trials in Health; University of Sheffield, Clinical Trials Research Unit<br>Morrison, Val; School of Psychology, Bangor University<br>Din, Nafees; Bangor University, North Wales Centre for Primary Care Research<br>Williams, Nefyn; Betsi Cadwaladr University Health Board, Research and Development; University of Liverpool, Department of Health Services Research

Keywords:	Process evaluation, Feasibility study, Hip fracture, Proximal femoral fracture, REHABILITATION MEDICINE, Self-efficacy

SCHOLARONE™
Manuscripts

Mixed methods process evaluation of an enhanced rehabilitation intervention for elderly hip fracture patients.

Jessica L Roberts PhD[1], Aaron W Pritchard MSc[2], Michelle Williams BSc[1], Nikki Totton MSc[1,3], Val Morrison PhD CPsychol[4], Nafees Ud Din MSc MFDS RCS[1], Nefyn H Williams PhD FRCGP[2,5.]

[1]School of Healthcare Sciences, Bangor University, UK

[2]Betsi Cadwaladr University Health Board, North Wales, UK

[3]School of Health and Related Research, Sheffield University, UK

[4]School of Psychology, Bangor University, UK

[5]Department of Health Services Research, University of Liverpool, UK

Corresponding author: Dr Jessica L Roberts, North Wales Centre for Primary Care Research, School of Healthcare Sciences, Bangor University, Cambrian 2, Wrexham Technology Park, Wrexham LL13 7YP, UK.

Tel: +44 (0)1248 383516 e-mail: j.l.roberts@bangor.ac.uk

Word count (3991)

For peer review only

Abstract (299 words)

Objectives: To describe the implementation of an enhanced rehabilitation programme in a randomised feasibility study compared with usual rehabilitation, and compare processes between the two; and to collect the views of patients, carers and therapy staff about trial participation.

Design: Mixed methods process evaluation in a randomised feasibility study

Setting: Patient participants were recruited on orthopaedic and rehabilitation wards; the intervention was delivered in the community following hospital discharge.

Participants: Sixty one older adults (aged ≥65) recovering from surgical treatment (replacement arthroplasty or internal fixation) following hip fracture, who were living independently prior to fracture and had mental capacity; and 31 of their carers.

Interventions: Usual care (control) or usual care plus an enhanced rehabilitation package (intervention). The enhanced rehabilitation consisted of a patient-held information workbook, goal-setting diary and up to six additional therapy sessions.

Process evaluation components: Recruitment of sites and rehabilitation teams, intervention delivery, response of rehabilitation teams, recruitment and reach in patient and carer participants, delivery to individuals, response of individual patients to the enhanced intervention or usual rehabilitation, response of carer participants, unintended consequences, testing intervention theory and context.

Results: Usual rehabilitation care was very variable. The enhanced rehabilitation group received a mean of five additional therapy sessions. All of the returned goal-setting diaries had inputs from the therapy team, and half had written comments by the patients and

For peer review only

carers. Focus group themes: variation of usual care and its impact on delivering the intervention; the importance of goal setting; the role of the therapist in providing reassurance about safe physical activities; acceptability of the extra therapy sessions. There were statistically significant correlations between three process measures of self-efficacy and disability/activities of daily living.

Conclusions: Lessons were learnt about delivering the enhanced rehabilitation intervention for a future definitive RCT.

Trial registration: ISRCTN22464643 Registered 21 July 2014

Key words: Process evaluation, feasibility study, hip fracture, proximal femoral fracture, rehabilitation, self-efficacy

Strengths and limitations of this study

- Mixed-methods process evaluation of a phase II randomised feasibility study which has examined recruitment, reach, dose delivered, intervention fidelity, unintended consequences, contextual factors and underlying theory.
- It will inform the delivery of a future, definitive, phase III RCT.
- It is not possible to comment on the longer-term implementation of the enhanced rehabilitation intervention, because this process evaluation was of a feasibility study with only a three month follow-up.
- The process data was analysed concurrently with the outcome data from the feasibility study, so the analysis of quantitative data was performed blind to treatment allocation; however, the qualitative findings and the feasibility study outcomes were discussed in relation to one another.

For peer review only

- Despite being a feasibility study with a small sample size, it was possible to gather some evidence supporting the underlying theory with regard to the importance of self-efficacy.

For peer review only

Background

Proximal femoral fracture, known as hip fracture, is a major health problem in the elderly [1], associated with a reduced ability to conduct activities of daily living independently [2]. Guidelines from the National Institute of Health and Care Excellence recommend the use of multidisciplinary rehabilitation programmes to maximise patient's recovery potential [3], but there is insufficient evidence of effectiveness and cost-effectiveness and the importance of individual components of these programmes in achieving desirable outcomes is poorly understood [4-6].

Study context

A study funded by the HTA programme [7] completed the first two phases of the MRC framework for complex interventions [8]. The first phase developed an enhanced rehabilitation intervention from the following working theory [9]:

"In the context of patients with a great range and variety of pre-fracture physical and mental comorbidities affecting their ability to meet rehabilitation goals, a tailored intervention incorporating increased amount of high quality practice of exercise and activities of daily living leads to better confidence, mood, function, mobility and reduced fear of falling."

In addition to usual care, the intervention included:

- Six home-based therapy sessions delivered by physiotherapists or occupational therapists with the assistance of a technical instructor providing reliable and consistent care.

For peer review only

- A novel, patient-held, workbook containing information on hip fracture, what to expect from rehabilitation, information about their role in their recovery, importance of physical activity and maintaining functional activities, and signposting to other services. The workbook contained a page of questions and Likert scale type response options to encourage participants to provide feedback on their workbook.
- A diary to facilitate patient-led goal setting, promote engagement and increase self-management.

A logic model described how the intervention components related to the programme theory [10].

The second phase of the study assessed the feasibility and acceptability of the new rehabilitation programme in a cohort study of all hip fracture patients with an embedded randomised feasibility study [10, 11]. This study assessed the feasibility of trial methods. Participants in the feasibility study were recruited from three acute hospitals across North Wales: East, Central and West. The rehabilitation intervention was delivered in the community. Participants were adults aged 65 years or older who had received surgical treatment for hip fracture, had been living independently prior to the hip fracture, had mental capacity as assessed by their clinical team, and received rehabilitation in the North Wales area. Between June 2014 and March 2015, 61 participants were equally randomised to usual care (control) or usual care plus the enhanced rehabilitation package (intervention). Compared with the anonymised cohort, they were younger, less likely to be re-admitted to hospital and less likely to die. Outcomes were measured at baseline and at three months' follow-up and included: disability, activities of daily living, anxiety and depression, health

For peer review only

utility, health service resource use, hip pain intensity, self-efficacy, fear of falling, physical function and carer strain.

Guidance from the UK Medical Research Council for developing and evaluating complex interventions recommends conducting a process evaluation, in order to 'explain discrepancies between expected and observed outcomes, and to provide insights to aid implementation' [8]. The aims of this process evaluation were to:

- Describe the implementation of the enhanced rehabilitation programme in the intervention group and usual rehabilitation in the control group;
- Describe and compare processes between the two forms of rehabilitation;
- Collect data from trial participants (patients, carers and therapy staff) about their experience of taking part in the trial;
- Collect data about contextual factors and test the theory underlying the intervention.

Methods

The study was influenced by Steckler and Linnan's process evaluation framework [12], and other proposed frameworks for designing and reporting process evaluations [13-15], other process evaluations of trials of complex interventions [16,17] as well as realist evaluation [18] (Table 1).

Mixed methods were used to collect process data. Routinely collected electronic health records using Therapy Manager software were used to extract usual rehabilitation activity data for participants in both intervention and control groups. The content of the additional enhanced rehabilitation sessions were recorded by therapy staff onto case report forms.

For peer review only

These described how the sessions were used for each patient, including the length of the session, where the session was delivered and the type of activities undertaken (Appendix 1). Workbooks and goal-setting diaries were collected from patients at follow-up and examined for degree of completion. Questionnaires completed by patients and carers contained health service resource use data. We used descriptive statistics to compare recruitment rates between the sites, and to describe the rehabilitation components used from the case report forms, routinely collected electronic records, and the completed workbooks and diaries.

We tested the theory underlying the intervention by testing the correlation between the main disability outcome measure (Barthel Index [19]) and three process measures of self-efficacy: General Self-Efficacy Scale (GSES) [20], Falls Self-Efficacy Scale – International (FES-I) [21,22] and Self-Efficacy for Exercise (SEE) [23]. The Barthel Index and GSES were collected at both baseline and follow-up, but FES-I and SEE were only completed at three month follow-up.

After the intervention was completed we carried out focus group interviews of patient and carer participants (Topic guide in Appendix 2). Separate focus groups were conducted for those in the control and intervention groups. Healthcare staff who delivered the intervention were also invited to separate focus groups at their nearest acute hospital site. Where staff were unable to attend, one-to-one telephone interviews were offered. Focus groups were recorded, transcribed and analysed thematically by two researchers. Patient and carers were asked about their experience of rehabilitation in general and of taking part in the FEMuR study.

For peer review only

Results

Recruitment of sites and rehabilitation teams

Rehabilitation team leads at three acute hospitals identified physiotherapists, based on the acute orthopaedic wards and occupational therapists and dual-trained technical instructors (TI) who were both acute and community based. The structure of the teams trained to deliver the intervention at each site differed depending on staff availability, with at least one Band 6 physiotherapist at each site who led the teams. As the physiotherapists in West and Central were based only within the acute hospital, the initial assessment session and introduction of the hip fracture workbook took place in the acute setting. In East, the physiotherapist was able to conduct this session with the patient in the community following discharge.

Intervention delivery

Data describing usual therapy care were only available from 35 participants recruited in the Central hospital, and associated community therapy teams, who were using Therapy Manager software. Five of these participants withdrew from the study and no further data regarding usual care were collected. Following discharge from the acute hospital, patients were discharged to their place of residence or for further rehabilitation in a community hospital prior to going home (Appendix 3). Ten patients had no details recorded relating to usual care following acute hospital discharge. Of the 20 patients who did have entries, four did not receive any face-to-face appointments with a healthcare professional, as their entries related to telephone calls to patients who were either uncontactable or declined

further treatment. For the 16 patients who received an appointment, there was a median of three appointments (n=4). The maximum number of appointments for one patient was 21. There was a total of 81 appointments for these 16 patients with 73 of these appointments (90%) conducted as home visits. Home visits were completed by different members of the therapy team (Appendix 4). If an assessment was required then a qualified physiotherapist (PT) or occupational therapist (OT) completed the visit, whilst subsequent visits following an agreed care plan were conducted by a technical instructor (TI). Most (90%) outpatient appointments were conducted by a physiotherapist (10% not recorded).

Activities in these usual rehabilitation sessions were categorised by the researcher as direct or indirect. Direct activities involved the practice of activities of daily living (25%), physical exercise (23%), phone calls with patients, discussion of progress and assessment of mood. Indirect activities were predominantly referrals to other services (33%), or contact with other members of the multidisciplinary team (30%).

Therapy Manager also recorded qualitative data. A number of patients were reluctant to engage in physical activity until they had been seen by a physiotherapist, even though in many cases they were told there would be a wait of at least four weeks.

Twenty-nine people were randomised to the enhanced rehabilitation intervention and details were available for 20 (reasons for missing data in Appendix 5). The majority (n=13) received all six sessions. The average number of sessions delivered was five, four of which took place in their own homes. One patient randomised to the intervention was discharged from the community hospital to a respite care home, so her intervention therapy sessions were delivered there.

For peer review only

TIs conducted the majority (55%) of intervention sessions, with 44% conducted by physiotherapists and the remaining 1% by more than one team member. The content of the intervention sessions depended upon individual patient need, at the discretion of the treating therapist. Therapists consistently completed the intervention paperwork detailing the types of activities and the time taken. Each session lasted approximately one hour, with an additional hour for travelling. In the intervention sessions, there was a lower rate of practising exercises (15%) and activities of daily living (14%) than usual care. Instead, there was more answering questions raised by the intervention workbook, working with goal setting diaries, giving feedback on progress and discussing emotional needs. For indirect activities, only 7% was used for discussion with the wider team and 4% for referring to other services. The remaining indirect activities included travel to appointments, writing notes, arranging further appointments and discussions with carers.

Response of rehabilitation teams

The initial recruitment period was planned to last six months. Due to staffing difficulties and the rurality of the West area, there were challenges delivering the intervention, and recruitment was slower than expected. Recruitment was extended for three months in Central and East, but was closed in the West.

Recruitment and reach in patient and carer participants

Rates of recruitment, eligibility and retention are given in Table 2. The main reasons for ineligibility were: lack of mental capacity 161 (49%), not living independently 61 (19%), younger than 65 years (13%), living out of area 30 (9%), treated without surgery 23 (7%). Patients were recruited after 193 (73%) eligible patients were approached with 176 (91%) of

For peer review only

these agreeing to talk to the researcher. Those who were not approached had either been: discharged home before recruitment, died, lived in areas where it was not possible to deliver the intervention, were deemed by clinical staff to be too ill to take part, or there were safety concerns that would have prevented the intervention being delivered due to lone worker policies. The main reasons for lack of recruitment in those approached were: burdensome 60 (31%), or disliked the study or questionnaire 13 (7%). Information concerning the number of visits it took to recruit participants were collected for 36 patients. The majority of patients had two visits, because recruitment occurred early in patients' recovery from surgery, and many requested a return visit to discuss the study after they had been discharged.

The retention rate was highest in the East and lowest in the West. The West encountered particular difficulties accessing staff for the trial, which might explain their poor retention rate. Nine patients withdrew from the study and four could not be contacted, so were lost to follow-up.

In addition, 41 carers were identified and 31 (75%) agreed to participate. Six carers withdrew from the study, and seven were lost to follow-up, leaving 18 (58%) who completed the follow-up questionnaire.

Delivery to individuals

Ten participants returned their goal-setting diaries and workbooks to the study team. All of the diaries had inputs from the therapists detailing the goals that were set in the initial assessment session. Five had also been updated by patients and their carers. These participants used the diaries extensively, updating their progress on the initial goals agreed

For peer review only

and entered by the therapist and including new goals, which they had entered into the diary themselves. Three of these participants also completed quizzes and hip fracture story sections of the workbook. One of the workbooks was completed by a carer who described the challenges to the patient's recovery and what they were doing to overcome them.

Response of individual patients to the enhanced intervention or usual rehabilitation

Four focus groups were conducted with patients and carers and two with healthcare professionals involved in the intervention (Table 3). Due to the geographical spread of participants in the West, it was not possible to conduct a focus group in this area, although one participant from this area was able to attend a focus group in Central. Healthcare professionals delivering the intervention in the West were unable to attend focus groups, but one acute physiotherapist and three technical instructors participated in individual telephone interviews. Four themes emerged:

Theme 1 - Variation of usual care and its impact on delivering the intervention

The frequency and format of usual community rehabilitation varied because of tailoring to individual need, the availability of resources and the provision of support services such as re-ablement and falls prevention classes. One carer described this variation as a "*postcode lottery*" (male carer, control group). In the control group, the initial contact with therapists often needed to be initiated by the patient, relying on their self-motivation, which was not necessary for the intervention group.

According to therapists, there was large geographical variation in usual care ranging from multiple same day appointments to no rehabilitation whatsoever. This variation affected how the enhanced rehabilitation intervention was delivered. One therapist commented that

when she delivered the intervention to patients with minimal usual care she would *"spread out the sessions, and then just pushed [the patient] harder, in the two weeks"* (physiotherapist). In contrast, where a comprehensive rehabilitation programme was available, she would deliver intervention sessions weekly in the confidence that at the end of the intervention period this provision would be continued through community run falls prevention or exercise schemes.

Therapists were concerned about supporting patients to set individualised goals when these might conflict with goals supported by other rehabilitation providers.

*"It was much harder when they had [another ongoing service], the re-ablement ones were much harder to actually, because somebody else was already setting what they were going to achieve" (*physiotherapist).

Theme 2 – The importance of goal-setting

Goal-setting was identified by therapists as playing an important role in engaging patients with their own recovery and in providing motivation to regain function and independence.

"I think [patients] probably more motivated because they can see the steps, to getting to that point. And why you are doing it" (physiotherapist).

The patient-held goal setting diary were appreciated by participants, as it gave them a direct focus and accountability for their goals.

*"You feel as if you have got a goal to get to, because you have put it in that book and you have got a goal." (*female patient, intervention group).

For peer review only

Therapists felt that the workbook and diary enabled patients to be more involved in their rehabilitation.

Theme 3 - The role of the therapist in providing reassurance about safe physical activities

The majority of patient participants reported anger and frustration when their physical ability to progress did not match their expectations, and they remained dependent on others.

"*Being incapacitated infuriated me so much"* (female patient, intervention group).

It was at this point that the physiotherapist played a pivotal role in managing expectations and reassuring patients that they were progressing normally. In the absence of this support there was a risk of patients losing motivation. This reassurance was important for giving patients the confidence to perform physical activities, as there was an underlying concern that they may otherwise do exercises which may be harmful.

"I had the security to know they were the right exercises, somebody there who gave them to me and you know they are qualified and they are telling you the right thing to do" (female patient, control group).

For participants in the control group, this lack of reassurance was a particular problem. Patients received a list of activities to avoid (hip precautions), but some were given no information about what exercises and activities were safe to perform, and wanted access to; "*somebody I could have just picked up the phone and said, how about this, should this be happening*" (female patient, control group).

For peer review only

Both groups identified this initial contact with therapists as vital for building their confidence and supporting their self-motivation for recovery.

"Once you have the information and the guidance on what to do, what not to do, I think we are intelligent enough to go away and do it, but it is just that initial guidance... we might be capable but you still need guidance" (male patient, control group).

Patients emphasised the importance of the intervention sessions in allowing them time to discuss their individual problems, particularly in the early stages of rehabilitation. This was facilitated by their relationship with the therapist or therapy team, where they felt comfortable enough to ask questions without fear of being dismissed or considered a nuisance. This was in contrast with how they felt in the acute hospital, or in usual care, where they were less informed about the processes and unfamiliar with the staff. A good relationship with their therapist underpinned successful rehabilitation, and enabled them to engage in, and take responsibility for, their role within the recovery process.

"I felt as though it was a sort of team effort, and she [the therapist] was sort of team leader, and knew what to do, and then it is sort of from part of the team if you like." (male patient, intervention group).

Theme 4 - Acceptability of the extra therapy sessions

Patient feedback on the intervention workbook varied. Some patients appreciated the explanation of the mechanics of their fracture and their better understanding of the surgery used to fix it.

"I thought it was good because it did explain things, it did explain to you what happens with a fracture" (female patient, intervention group).

For peer review only

Other patients reflected on the comfort that this additional information gave them.

"I didn't know what to expect but I found I read [the workbook] profusely every day, and I did, I found it very, very helpful. It made me feel that I wasn't on my own" (female patient, intervention group).

Other participants found the workbooks less useful.

"I sort of read it once and thought well you know this isn't very useful" (male patient, intervention group).

Without exception, the most useful aspect of the intervention was considered to be the extra time that participants received with the therapy teams. The goal-setting diary and information workbook were seen as useful supporting documents to these extra sessions. Whilst therapists acknowledged the complex nature of delivering intervention sessions in an environment of varied usual care, it was generally accepted that the extra sessions were a great benefit to patients. The analysis of the focus groups also led to the development of the GUIDE tool (Figure 1) which summarises the role of the therapist in the rehabilitation process, incorporating important factors identified by patients and their carers.

Unintended consequences

There were nine adverse events, six were serious, two resulted in re-admission to the acute hospital and there was one death; none were related to participation in the study.

Testing the intervention theory

Correlations between the Barthel Index measuring disability/independence in activities of daily living and the three process measures of self-efficacy were statistically significant and

For peer review only

suggested that as the participants' activities of daily living increased, so did their self-efficacy (Appendix 6). Higher scores in the FES-I represent a greater fear of falling, so the relationship showed that as the fear of falling increased, activities of daily living decreased. The strongest correlation was with the FES-I.

Discussion

Summary of main findings

This trial took place in three sites across North Wales. Recruitment to the trial was more difficult in the West because of its rurality and also staff shortages. The recruitment rate was highest in Central; the retention rate was highest in the East. Usual rehabilitation care was very variable with a median of three appointments; the enhanced rehabilitation group received a mean of five additional therapy sessions. Variation in usual care affected how the enhanced rehabilitation intervention was delivered. TIs carried out most of the sessions, which consisted of practising exercises and activities of daily living, goal-setting, answering questions raised by the workbook and giving feedback on progress. Goal-setting had an important role in engaging patients in their own recovery, which was assisted by the workbook and diary. All of the returned goal-setting diaries had inputs from the therapy team, and half had written comments by the patients and carers. Some participants did not find the workbook and diary useful, but all valued the extra therapy sessions. The physiotherapist was very important for managing patients' expectations and for reassuring them about what physical activity was safe to perform. The lack of reassurance was particularly problematic for some in the control group. There were statistically significant

For peer review only

correlations between three process measures of self-efficacy and activities of daily living, which supported the theory underlying the intervention.

Strengths and weaknesses

This was a mixed-methods process evaluation performed concurrently with a randomised feasibility study which has examined recruitment, reach, dose delivered, intervention fidelity, unintended consequences, contextual factors and underlying theory. It provides further insights into how to deliver the intervention for a future, definitive, phase III RCT. Although many commented that goal-setting was enhanced by the workbook and self-monitoring diaries, half of the collected workbooks were not completed, and some found them unhelpful. Feedback from participants and intervention delivery staff will result in further refinement of the workbook and diary and also the development of training materials before the planned definitive trial. Because this process evaluation was only part of a feasibility study it is not possible to comment on longer-term implementation of the enhanced rehabilitation intervention. The process data was analysed concurrently with the outcome data from the feasibility study, so the analysis of quantitative data was performed blind to treatment allocation; however, the qualitative findings and the feasibility study outcomes were discussed in relation to one another. It was not possible to collect data on usual rehabilitation from all participants in the intervention group, because the Therapy Manager software was only used by the rehabilitation teams in the Central area. Despite being a feasibility study with a small sample size, it was possible to gather some evidence supporting the underlying theory with regard to the importance of self-efficacy.

Comparison with previous literature

For peer review only

Qualitative interviews of participants in the Exercise-Plus RCT in the United States, of a motivational intervention designed to increase adherence to rehabilitation exercise also found that identifying goals and improving self-efficacy were important, and an exercise booklet provided useful visual cues [24]. A good relationship with the therapist providing individualised care and verbal encouragement resulted in participants reciprocating their therapists' kindness by completing the exercise programme. They also described constraints to exercise such as unpleasant sensations of pain and fatigue, lack of time and space, and discontinuing their exercise once baseline function was restored. A qualitative study of a rehabilitation programme in Taiwan found that when therapists emphasised social support and resilience, patients developed more self-confidence and independence [25]. A process evaluation of a rehabilitation intervention in Sweden found that hip fracture had long lasting 'social and existential' effects on patients necessitating both physical and emotional support during recovery [26].

Implications for a future definitive RCT

Several lessons have been learnt for delivering the enhanced rehabilitation intervention for a definitive phase III RCT (Box 1). Recruitment was harder in rural areas, especially in areas with staff shortages, which will be an important consideration when choosing sites for the definitive trial. Research staff need to remain flexible, be prepared to recruit after discharge home, keep visiting potential participants and possibly delay recruitment until later after surgery. Employing therapists directly by the research team or secondment from the research delivery workforce would avoid them being pulled back to clinical work during staffing shortages. The workbook and goal-setting diary need to be refined further in the light of feedback from patients, carers and clinicians. Finally, a mnemonic (GUIDE) for

For peer review only

therapists has been developed following the qualitative research (Figure 1), which will be useful as a training tool for the therapy teams prior to a definitive phase III RCT.

Acknowledgments

The FEMuR team would like to thank all participants who took part in the study. The authors would also like to thank Health and Care Research Wales for supporting participant recruitment, and the healthcare staff at BCUHB who identified potential participants and delivered the intervention. NT institution affiliation changed from Bangor University to University of Sheffield during the study.

Contributors

NHW was the chief investigator and grant holder, was responsible for study design, conduct and analysis and had overall responsibility for the study and acts as guarantor. JLR was the study manager overseeing day-to-day conduct, participant recruitment, methodological input, conducted qualitative analysis for the focus groups. NUD was involved in participant recruitment, acquisition of quantitative and qualitative data and analysis. NT conducted the statistical analysis for the feasibility study. VM was a coinvestigator responsible for study design, provided health psychology expertise and methodological oversight throughout the study. MW was involved in the conduct of the study including maintenance of trial documentation, acquisition of data, provided administrative support. AWP contributed to qualitative analysis of the focus groups. All authors were involved in drafting, revising and approving this manuscript.

Competing Interests

For peer review only

NHW, JLR, NUD, MW, NT, VM report a grant from NIHR HTA programme, for the conduct of the study. NHW reports additional grants from Public Health Wales, NIHR HTA and BCUHB, outside the submitted work.

Funding statement

This work was supported by the National Institute for Health Research's Health Technology Assessment Programme, grant number 11/33/03. The views and opinions expressed therein are those of the authors and do not necessarily reflect those of the HTA, NIHR, NHS or the Department of Health.

Ethics approval

The study received ethical approval from the UK NHS North Wales West Research Ethics Committee—West. Ref 13/WA/0402 and NHS Research and Development approval from the Betsi Cadwalader University Health Board (BCUHB) Internal Review Panel.

Availability of data and materials

The datasets used during the current study are available from the corresponding author on reasonable request.

References

1. Johansen A, et al., National Hip Fracture Database. National report 2013, C.E.a.E. Unit, Editor 013, Royal College of Physicians: London.

For peer review only

2. Bertram, M., et al., *Review of the long-term disability associated with hip fractures.* Injury Prevention, 2011. **17**(6): p. 365-370.

3. National Clinical Guideline Centre. Hip fracture: the management of hip fracture in adults| Guidance and guidelines | NICE. National Institute of Health and Care Excellence 2011. Accessed 2017. Available at [*http://www.nice.org.uk/guidance/cg124*](http://www.nice.org.uk/guidance/cg124).

4. Handoll, H.H., et al., *Multidisciplinary rehabilitation for older people with hip fractures.* Cochrane Database of Systematic Reviews, 2009(4).

5. Handoll, H.H., C. Sherrington, and J.C. Mak, *Interventions for improving mobility after hip fracture surgery in adults.* Cochrane Database of Systematic Reviews, 2011(3).

6. Crotty, M., et al., *Rehabilitation interventions for improving physical and psychosocial functioning after hip fracture in older people.* Cochrane Database of Systematic Reviews, 2010(1).

7. Williams NH, Roberts JL, Din NU, Charles JM, Totton N, Williams M, Mawdesley K, Hawkes CA, Morrison V, Lemmey A, Edwards RhT, Hoare Z, Pritchard AW, Woods RT , Alexander S, Sackley C, Logan P, Wilkinson C, Rycroft-Malone J. A multidisciplinary rehabilitation package following hip fracture: Fracture in the Elderly Multidisciplinary Rehabilitation (FEMuR). Health Technol Assess 2017; 21 (44).

8. Medical Research Council. Developing and evaluating complex interventions: new guidance. London: Medical Research Council, 2008.

9. Roberts JL, Din NU, Williams M, Hawkes CA, Charles JM, Hoare Z, Morrison V, Alexander S, Lemmey A, Sackley C, Logan P, Wilkinson C, Rycroft-Malone J, Williams NH. Development of an evidence-based complex intervention for community rehabilitation of hip fracture patients using realist review, survey and focus group. BMJ Open 2017; 7: e014362.

For peer review only

10. Williams NH, Hawkes C, Din NU, Roberts J, Charles JM, Morrison V, Hoare Z, Edwards RhT, Andrew G, Alexander S, Lemmey A, Woods B, Sackley C, Logan P, Hunnisett D, Mawdesley K, Wilkinson C. Fracture in the Elderly Multidisciplinary Rehabilitation (FEMuR): study protocol for a Phase II randomised feasibility study of a multidisciplinary rehabilitation package following hip fracture [ISRCTN22464643] Pilot feasibility Studies 2015; 1: 13.

11. Williams NH, Roberts JL, Din NU, Totton N, Charles JM, Hawkes CA, Morrison V, Hoare Z, Williams M, Pritchard AW, Mawdesley K, Hawkes CA, Morrison V, Lemmey A, Edwards RhT, Hoare Z, Pritchard AW, Alexander S, Lemmey A, Woods RT, Sackley C, Logan P, Edwards RhT, Wilkinson C. Fracture in the Elderly Multidisciplinary Rehabilitation (FEMuR): a phase II randomised feasibility study of a multidisciplinary rehabilitation package following hip fracture. BMJ Open 2016; 6: e012422.

12. Steckler A, Linnan L (Eds.) Process evaluation for public health interventions and research. San Francisco: Jossey-Bass, 2002.

13. Moore G, Audrey S, Barker M, Bond L, Bonell C, Hardeman W, Moore L, O'Cathain A, Tinati T, Wight D, Baird J. Process evaluation of complex interventions: UK Medical Research Council (MRC) Guidance. MRC Population Health Science Research Network, 2014.

14. Mastersson-Allgar P. Advancing process evaluation research within the field of Neurological rehabilitation. PhD thesis, Bangor University, 2016.

15. Grant A, Treweek S, Dreischulte T, Foy R, Guthrie B. Process evaluations for cluster randomised trials of complex interventions: a proposed framework for designing and reporting. Trials 2013; 14: 15.

16. Oakley A, Strange V, Bonell C, Allen E, Stephenson J, RIPPLE Study Team. Process evaluation in randomised controlled trials of complex interventions. BMJ 2006; 332: 413-

For peer review only

416.

17. Moore GF, Raisanen L, Moore L, Din NU,Murphy S. Mixed-method process evaluation of the Welsh National Exercise Referral Scheme. Health Education 2013; 113(6): 476-501.

18. Pawson R, Tilley N. Realistic evaluation, London: Sage, 1997.

19. Mahoney F, Barthel D. Functional evaluation: the Barthel index. Maryland State Med J. 1965;14:56–61.

20. Schwarzer R, Jerusalem M. Generalized self-efficacy scale. In: Weinman J, Wright S, Johnston M, editors. Measures in health psychology: a user's portfolio causal and control beliefs. Windsor, UK: NFER-NELSON; 1995. p. 35–7.

21. Hauer K, Yardley L, Beyer N, Kempen G, Dias N, Campbell M, et al. Validation of the Falls Efficacy Scale and Falls Efficacy Scale International in geriatric patients with and without cognitive impairment: results of self-report and interview-based questionnaires. Gerontology. 2010;56:190–9.

22. Yardley L, Beyer N, Hauer K, Kempen G, Piot-Ziegler C, Todd C. Development and initial validation of the Falls Efficacy Scale-International (FES-I). Age Ageing. 2005;34:614–9.

23. Resnick B, Jenkins LS. Testing the reliability and validity of the self-efficacy for exercise scale. Nurs Res. 2000;49:154–9.

24. Resnick B, Orwig D, Wehren L, Zimmerman S, Simpson M, Magaziner J. The Exercise Plus program for older women post hip fracture: participant perspectives. Gerontologist. 2005;45:539–44.

25. Huang T, Acton G. Ways to maintain independence among Taiwanese elderly adults with hip fractures: A qualitative Study**.** Geriatric Nurs. 2009;30:28-35

26. Ziden L, Wenestam C, Hansson-Scherman M. A life-breaking event: early experiences of the consequences of a hip fracture for elderly people. Clin Rehab. 2008;22:801–11**.**

For peer review only

Table 1 Process evaluation questions and methods for evaluating

Component	Process evaluation questions	Research methods	Stage of study to collect data
Recruitment of sites and rehabilitation teams	How are sites and teams recruited?	Documentation of recruitment process by research team	Pre-intervention
	Which sites and teams agree to participate?	Quantitative comparison of recruited and non-recruited sites	
Intervention delivery	What rehabilitation intervention is delivered? Is it what was intended by the researchers?	Quantitative examination of case report forms and of electronic data entered onto Therapy Manager software	During intervention
Response of rehabilitation teams	How is the enhanced intervention adopted by the rehabilitation teams	Quantitative examination of case report forms and qualitative interviews of rehabilitation team members	During and following the intervention
Recruitment and reach in patient and carer participants	How many are recruited into the feasibility study? Are they representative?	Quantitative comparison between feasibility study and anonymised cohort	During the intervention
	Who is recruited into the feasibility study? What are the reasons for non-recruitment?	Examination of recruitment log	During the intervention
Delivery to individuals	What intervention is delivered to each participant?	Quantitative examination of case report forms and of electronic data entered onto Therapy Manager software	During the intervention
	Is the delivered intervention the one intended by the researchers?	Measurement of intervention fidelity: completion of workbook tasks, completion of diaries, number and content of therapy sessions	During the intervention
Response of individual patients to the enhanced intervention or usual rehabilitation	How do the patient participants respond?	Qualitative analysis of focus group data about patient participants' experience and response to the intervention and to usual care	Following the intervention
Response of carer participants	Effects on carers	Qualitative analysis of focus group data about carers' experiences	Following the intervention
Unintended consequences	Are there unintended changes in processes and outcomes related to the intervention and unrelated to care?	Quantitative examination of adverse effects reports, health service activity data from patient completed questionnaires and routinely collected electronic sources. Qualitative analysis of focus group data from patients and	During and following the intervention

For peer review only

		their carers	
Theory	What theory has been used to develop the intervention?	Quantitative data analysis of process outcome measures to assess predicted relationships	Following the intervention
Context	What is the wider context in which the feasibility study is conducted?	Realist review of the rehabilitation literature, survey of current rehabilitation practice, focus groups of patients, carers and rehabilitation professionals. Quantitative comparison with anonymised cohort	Pre-intervention (phase I study) and during the intervention

For peer review only

Table 2 Eligibility, recruitment and retention rates according to acute hospital site

Number of Patients	West	Central	East	Total
**Screened (rate)**	147	235	211	593
**Eligible (rate)**	75 (51%)	103 (44%)	88 (42%)	266 (45%)
**Recruited (rate)**	11 (15%)	35 (34%)	16 (18%)	62 (23%)
**Retained (rate)**	4 (36%)	29 (83%)	16 (100%)	49 (79%)

For peer review only

Table 3 Focus group participants' characteristics

Participant type	Location	Attendees
Patient and carers in control group	East	2 female patients, 1 male patient, 2 male carers (n=5)
Patient and carers in control group	Central	2 female patients, 1 male patient, 1 female carer (n=4)
Patient and carers in intervention group	East	3 female patients (n=3)
Patient and carers in intervention group	Central	2 male patients, 2 female patients, 1 male carer, 2 female carers (n=7)
Healthcare professionals	East	Clinical specialist physiotherapist, 2 orthopaedic physiotherapists, physiotherapy technical instructor (n=4)
Healthcare professionals	Central	Orthopaedic acute physiotherapist, rotational physiotherapist, physiotherapy technical instructor (n=3)

For peer review only

Box 1 Ten lessons learnt for a future definitive randomised controlled trial

Sites	1	Consider staff availability and rurality when recruiting sites
Therapy staff	2	Consider employing therapy staff directly or second from research delivery teams
Participant recruitment	3	Recruitment flexibility – including after discharge home
	4	Keep visiting potential participants
	5	Delay recruitment until later after surgery
Trial-specific training	6	Use the mnemonic GUIDE as a training aid
	7	Stress the importance of the first home visit to reassure participants about safe activities
	8	Value the emotional support provided by the technical instructors
Extra therapy sessions	9	Allow some participants to have more than six extra sessions
Workbook	10	Further refine the workbook in the light of feedback

For peer review only

Figure Legends

Figure 1. GUIDE mnemonic for therapists involved in rehabilitation following hip fracture

For peer review only

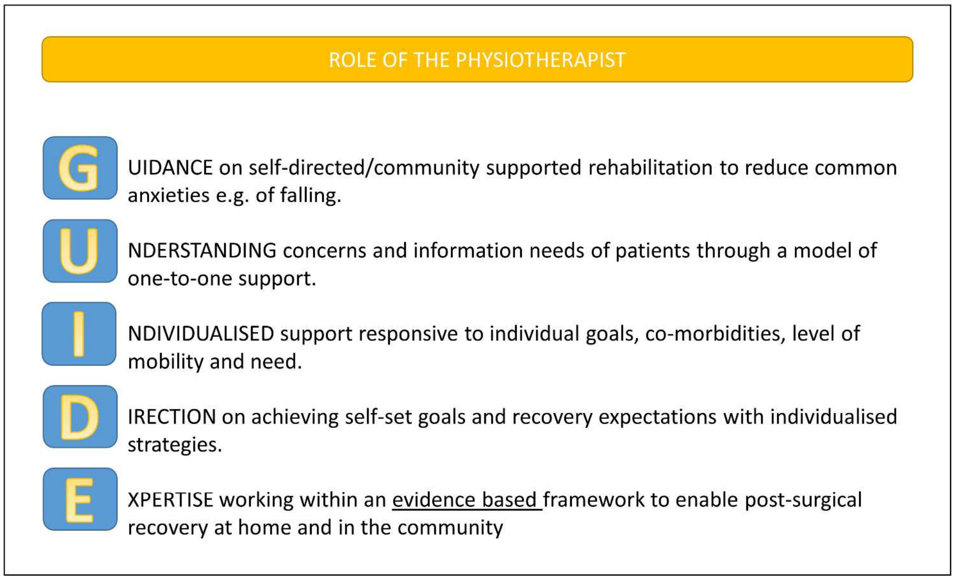

Figure 1. GUIDE mnemonic for therapists involved in rehabilitation following hip fracture

275x190mm (300 x 300 DPI)

For peer review only - http://bmjopen.bmj.com/site/about/guidelines.xhtml

Appendix 1 Case report form

Session number:

Patient Study ID: ☐☐☐☐☐

Date: ____ /____ /________ **Total amount of time used for this session:** ____

Role(s) of person/people conducting visit: Occupational therapist / Physiotherapist / OT assistant / Physiotherapy assistant / Technical instructor / Other

Location of session: Patient's own home / Care home where patient lives permanently / Community hospital outpatients / Acute hospital outpatients / ________________________ Other

<u>1. Please indicate what ***indirect*** care activities you carried out for the patient during this session, and for how long:</u>

**Activity**	**Time (e.g. 10 minutes)**	**Further details**
Referring to another service		
Phone contact with patient		
Contact with MDT about patient		
Patient admin		
Travel to and from session		
Other, please specify.		

<u>2. Please indicate what ***direct*** care activities you carried out for the patient during this session, and for how long:</u>

**Activity**	**Time (e.g. 20 minutes)**	**Further details**
Assessment/re-assessment		
Physical exercises		
Activities of daily living practice		
Workbook		
Discussion of care and progress		

For peer review only

Activity	Time (e.g. 20 minutes)	Further details
Discussion of referral to a follow on service e.g. social groups, falls groups, outpatients physio		
Travel to and from session		
Other, please specify		

3. If you spent time on the ***workbook*** in the session, please indicate what this involved:

Activity	Time (e.g. 5 minutes)	Further details
Goal setting		
Reviewing progress		
Answering patient questions stimulated by workbook		
Emotional support		
Other, please specify		

For peer review only

Appendix 2 Focus group topic guides

Patient and carer topic guide – control group

- What was your experience of the rehabilitation you received after your hip fracture, specifically regarding physiotherapy and exercise?
- What went well, what could be improved? Having experienced fracture and the rehabilitation involved, what would you recommend to improve care?
- If there are any attending the focus group, what were the experiences of participants who were discharged to nursing/residential care, or those who were more dependent on carers?

- Did your therapists give you exercises to do?
  - How confident did you feel about practising them?
  - Did your confidence change over time? What changed this?
- Were you given any written information to keep with you?
  - How helpful was this? Was there any other information you would have found useful?
- How involved did you feel in planning your recovery and rehabilitation?
- Often people say they were afraid of falling again after having a hip fracture, did you experience this?
  - Was there anything in your rehabilitation that helped reduce the fear of falling?
  - Did you attend a falls prevention class? How did you find out about this and was it useful?
- How did you feel about being approached to take part in the study?
  - Did you understand the information you were given?

For peer review only

- What made you decide to take part?
- How did you feel about the questions you were asked in the hospital and at the follow up, especially about how long they took and how well you were able to understand the questions and their relevance?

- How did you feel about not knowing which study group you would be in when you agreed to take part?
  - How would you have felt if you had been in the other group?
- Did you do some exercise tests with your community physiotherapists at the end of the study?
  - How did you feel about being asked to do these?
- Has anything changed for you as a consequence of being in the study?
  - If you were asked to take part now, would you make the same decision?

Patient and carer topic guide – intervention group

- What was your experience of the rehabilitation you received after your hip fracture?
  - What went well, what could be improved?
  - If there are any attending the focus group, what were the experiences of participants who were discharged to nursing/residential care, or those who were more dependent on carers?
- TASK - Rank the following aspects of the intervention according to how useful they were:
  - Extra time with a therapist
  - Information in the workbook
  - Being involved in setting your own goals

For peer review only

  - Keeping a diary to look at progress
- Discussion of ranking
- Did you feel confident to suggest goals to your therapists and how did that help you?
- What did you like best about the workbook?
  - Was there anything you felt the workbook was missing?
- Were you aware of the extra sessions which you received because you were in the study?
  - Were these sessions used differently to the care as usual sessions?
  - Did you notice any improvements in your recovery as a result of this extra time?
- Did your therapists give you exercises to do?
  - How confident did you feel about practising them?
  - Did your confidence change over time? What changed this?
- Often people say they were afraid of falling again after having a hip fracture, did you experience this?
  - Was there anything in your rehabilitation that helped reduce the fear of falling?
  - Did you attend a falls prevention class? How did you find out about this and was it useful?
- How did you feel about being approached to take part in the study?
  - Did you understand the information you were given?
  - What made you decide to take part?
  - How did you feel about the questions you were asked in the hospital and at the follow up, especially about how long they took and how well you were able to understand the questions and their relevance?

For peer review only

- Did you do some exercise tests with your community physiotherapists at the end of the study?
    - How did you feel about being asked to do these?
- How did you feel about not knowing which study group you would be in when you agreed to take part?
    - How would you have felt if you had been in the other group?
- Has anything changed for you as a consequence of being in the study?
    - If you were asked to take part now, would you make the same decision?

Healthcare professional topic guide

- Experiences of the enhanced rehabilitation intervention
    - What went well?
    - What could be improved?
- Experiences of extra therapist time
- Awareness of extra time available to patients in the study
    - How did if work, what went well what needs to be improved
    - How did you use the extra time with patients? Do you think it made a difference?
- Views of the Workbook
    - Did you use it?
    - If so how, what was useful, what could be improved, was anything missing you think would have been helpful?

For peer review only

- Do you feel the intervention made any difference to the way patients engaged with you and participated in their rehabilitation?
    - If so how/if not why?
    - What was your experience the goal setting and feedback in the work book? Similar/different to usual way you work/good points/areas for improvement
    - What was your experience of the information sections? Useful to you/useful to patients/anything you weren't aware of before/ anything that was missing?
- Experiences of working with patients with cognitive impairments and their carers
    - How did the intervention go?
- Experiences of working with patients who were discharged to long term residential/nursing care
    - How did the intervention go?
- Feasibility of physiotherapists conducting the physical measures at 3 month follow-up
- Acceptability of the outcome measures for patients
    - Timing; Number; Content
- Anything else we haven't talked about you think has been important in working with this intervention?

For peer review only

Appendix 3 Rehabilitation pathways in usual care on discharge from the central acute hospital following surgical repair of hip fracture

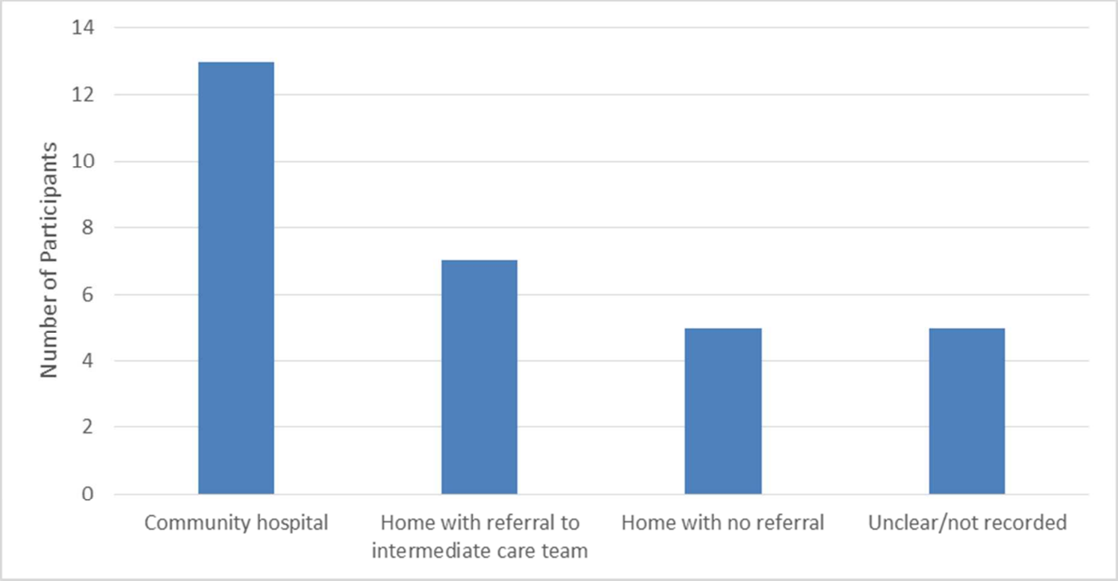

Appendix 4 Percentage of home visits conducted by different members of the rehabilitation team for usual care

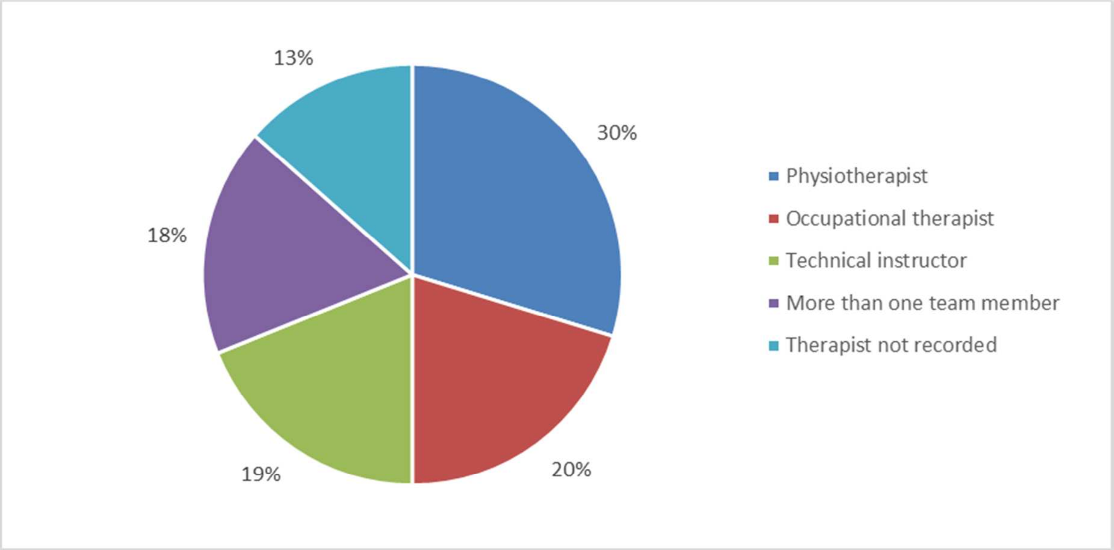

For peer review only

Appendix 5 Reasons for missing data on intervention session use

Reason	No. of participants	Area
Withdrawn	4	Gwynedd, Anglesey, Conwy and Denbighshire
Area not covered by therapist	1	Gwynedd and Anglesey
Declined intervention sessions	1	Conwy and Denbighshire
Could not be contacted to arrange sessions	1	Conwy and Denbighshire
Received intervention but therapist not returned paperwork	1	Gwynedd and Anglesey
Therapist delivering sessions moved to different area and could not complete intervention	1	Gwynedd and Anglesey

For peer review only

For peer review only

Appendix 6 Correlation analysis of Barthel Index with measures of self-efficacy

Self-Efficacy Measures	Barthel Index at baseline<br>Correlation (95% Confidence Intervals)	Barthel Index at 3 months<br>Correlation (95% Confidence Intervals)
General Self-Efficacy Scale (Baseline and 3 Months)	r_{55}= 0.43 (0.12 to 0.68), p=0.001	r_{37}= 0.59 (0.23 to 0.82), p<0.001
Falls Efficacy Scale – International* (3 Months)	Not Collected	r_{34}= -0.68 (-0.83 to -0.45), p<0.001
Self-Efficacy for Exercise Scale (3 Months)	Not Collected	r_{33}= 0.62 (0.26 to 0.82), P<0.001

* High values represent high levels of fear of falling

For peer review only

Mixed methods process evaluation of an enhanced community-based rehabilitation intervention for elderly hip fracture patients.

Journal:	*BMJ Open*
Manuscript ID	bmjopen-2018-021486.R1
Article Type:	Research
Date Submitted by the Author:	15-May-2018
Complete List of Authors:	Roberts, Jessica; Bangor University, North Wales Centre for Primary Care Research<br>Pritchard, Aaron; Betsi Cadwaladr University Health Board, Research and Development<br>Williams, Michelle; Bangor University<br>Totton, Nikki; Bangor University College of Health and Behavioural Sciences, North Wales Organisation for Randomised Trials in Health; University of Sheffield, Clinical Trials Research Unit<br>Morrison, Val; School of Psychology, Bangor University<br>Din, Nafees; Bangor University, North Wales Centre for Primary Care Research<br>Williams, Nefyn; Betsi Cadwaladr University Health Board, Research and Development; University of Liverpool, Department of Health Services Research
**Primary Subject Heading**:	Rehabilitation medicine
Secondary Subject Heading:	Health services research, Qualitative research, Research methods, Geriatric medicine
Keywords:	Process evaluation, Feasibility study, Hip fracture, Proximal femoral fracture, REHABILITATION MEDICINE, Self-efficacy

Mixed methods process evaluation of an enhanced community-based rehabilitation intervention for elderly hip fracture patients.

Jessica L Roberts PhD[1], Aaron W Pritchard MSc[2], Michelle Williams BSc[1], Nikki Totton PhD[3], Val Morrison PhD CPsychol[4], Nafees Ud Din MSc MFDS RCS[1], Nefyn H Williams PhD FRCGP[2,5.]

[1]School of Healthcare Sciences, Bangor University, UK

[2]Betsi Cadwaladr University Health Board, North Wales, UK

[3]School of Health and Related Research, Sheffield University, UK

[4]School of Psychology, Bangor University, UK

[5]Department of Health Services Research, University of Liverpool, UK

Corresponding author: Dr Jessica L Roberts, North Wales Centre for Primary Care Research, School of Healthcare Sciences, Bangor University, Cambrian 2, Wrexham Technology Park, Wrexham LL13 7YP, UK.

Tel: +44 (0)1248 383516 e-mail: j.l.roberts@bangor.ac.uk

For peer review only

Abstract

Objectives: To describe the implementation of an enhanced rehabilitation programme in a randomised feasibility study compared with usual rehabilitation, and compare processes between the two; and to collect the views of patients, carers and therapy staff about trial participation.

Design: Mixed methods process evaluation in a randomised feasibility study

Setting: Patient participants were recruited on orthopaedic and rehabilitation wards; the intervention was delivered in the community following hospital discharge.

Participants: Sixty one older adults (aged ≥65) recovering from surgical treatment (replacement arthroplasty or internal fixation) following hip fracture, who were living independently prior to fracture and had mental capacity; and 31 of their carers.

Interventions: Usual care (control) or usual care plus an enhanced rehabilitation package (intervention). The enhanced rehabilitation consisted of a patient-held information workbook, goal-setting diary and up to six additional therapy sessions.

Process evaluation components: Recruitment of sites and rehabilitation teams, response of rehabilitation teams, recruitment and reach in patient and carer participants, intervention delivery, delivery to individuals, response of individual patients to the enhanced intervention or usual rehabilitation, response of carer participants, unintended consequences, testing intervention theory and context.

Results: Usual rehabilitation care was very variable. The enhanced rehabilitation group received a mean of five additional therapy sessions. All of the returned goal-setting diaries had inputs from the therapy team, and half had written comments by the patients and

For peer review only

carers. Focus group themes: variation of usual care and its impact on delivering the intervention; the importance of goal setting; the role of the therapist in providing reassurance about safe physical activities; acceptability of the extra therapy sessions.

Conclusions: Lessons learnt for a future definitive RCT include how to enhance recruitment, and improve training materials, the workbook, delivery of the extra therapy sessions and recording of usual rehabilitation care.

Trial registration: ISRCTN22464643 Registered 21 July 2014

Key words: Process evaluation, feasibility study, hip fracture, proximal femoral fracture, r

Strengths and limitations of this study

- Mixed-methods process evaluation of a phase II randomised feasibility study which has examined recruitment, reach, dose delivered, intervention fidelity, unintended consequences, contextual factors and underlying theory.
- It will inform the delivery of a future, definitive, phase III RCT.
- It is not possible to comment on the longer-term implementation of the enhanced rehabilitation intervention, because this process evaluation was of a feasibility study with only a three month follow-up.
- The process data was analysed concurrently with the outcome data from the feasibility study, so the analysis of quantitative data was performed blind to treatment allocation; however, the qualitative findings and the feasibility study

For peer review only

- Despite being a feasibility study with a small sample size, it was possible to gather some evidence supporting the underlying theory with regard to the importance of self-efficacy.

For peer review only

Background

Proximal femoral fracture, known as hip fracture, is a major health problem in the elderly [1], associated with a reduced ability to conduct activities of daily living independently [2]. Guidelines from the National Institute of Health and Care Excellence recommend the use of multidisciplinary rehabilitation programmes to maximise patient's recovery potential [3], but there is insufficient evidence of effectiveness and cost-effectiveness and the importance of individual components of these programmes in achieving desirable outcomes is poorly understood [4-6].

Study context

A study funded by the HTA programme [7] completed the first two phases of the MRC framework for complex interventions [8]. The first phase developed an enhanced rehabilitation intervention from the following working theory [9]:

"In the context of patients with a great range and variety of pre-fracture physical and mental comorbidities affecting their ability to meet rehabilitation goals, a tailored intervention incorporating increased amount of high quality practice of exercise and activities of daily living leads to better confidence, mood, function, mobility and reduced fear of falling."

In addition to usual care, the intervention included:

- Six home-based therapy sessions delivered by physiotherapists or occupational therapists with the assistance of technical instructor providing reliable and consistent care.

For peer review only

- A novel, patient-held, workbook containing information on hip fracture, what to expect from rehabilitation, information about their role in their recovery, importance of physical activity and maintaining functional activities, and signposting to other services. The workbook contained a page of questions and Likert scale type response options to encourage participants to provide feedback on their workbook.
- A diary to facilitate patient-led goal setting, promote engagement and increase self-management.

A logic model described how the intervention components related to the programme theory [10].

The second phase of the study was a randomised feasibility study, which assessed the acceptability of the new rehabilitation programme and the feasibility of trial methods [10, 11]. Participants in the feasibility study were recruited from three acute hospitals across North Wales: East, Central and West. The rehabilitation intervention was delivered in the community. Participants were adults aged 65 years or older who had received surgical treatment for hip fracture, had been living independently prior to the hip fracture, had mental capacity as assessed by their clinical team, and received rehabilitation in the North Wales area. Between June 2014 and March 2015, 61 participants were randomised to usual care (control) or usual care plus the enhanced rehabilitation package (intervention). The mean Abbreviated Mental Test Score for these participants was 9.1 (standard deviation 1.3 and range 5 to 10). A score of 8 or less suggests cognitive impairment [12].

In addition, participants recruited to the feasibility study were compared with an anonymised cohort of all patients admitted to the same acute hospitals with a proximal femoral fracture over a similar time period. Compared with this cohort, the study

For peer review only

participants were younger, less likely to be re-admitted to hospital and less likely to die. Outcomes were measured at baseline and at three months' follow-up and included: disability, activities of daily living, anxiety and depression, health utility, health service resource use, hip pain intensity, self-efficacy, fear of falling, physical function and carer strain.

Guidance from the UK Medical Research Council for developing and evaluating complex interventions recommends conducting a process evaluation, in order to 'explain discrepancies between expected and observed outcomes, and to provide insights to aid implementation' [8]. The aims of this process evaluation were to:

- Describe the implementation of the enhanced rehabilitation programme in the intervention group and usual rehabilitation in the control group;
- Describe and compare processes between the two forms of rehabilitation;
- Collect data from trial participants (patients, carers and therapy staff) about their experience of taking part in the trial;
- Collect data about contextual factors and test the theory underlying the intervention.

Methods

The study was influenced by Steckler and Linnan's process evaluation framework [13], and other proposed frameworks for designing and reporting process evaluations [14-16], other process evaluations of trials of complex interventions [17,18] as well as realist evaluation [19] (Table 1).

For peer review only

Mixed methods were used to collect process data. Routinely collected electronic health records using Therapy Manager software were used to extract usual rehabilitation activity data for participants in both intervention and control groups. The content of the additional enhanced rehabilitation sessions were recorded by therapy staff onto case report forms. These described how the sessions were used for each patient, including the length of the session, where the session was delivered and the type of activities undertaken (Appendix 1). Workbooks and goal-setting diaries were collected from patients at follow-up and examined for degree of completion. Questionnaires completed by patients and carers contained health service resource use data. We used descriptive statistics to compare recruitment rates between the sites, and to describe the rehabilitation components used from the case report forms, routinely collected electronic records, and the completed workbooks and diaries.

We tested the theory underlying the intervention by testing the correlation between the main outcome measure (Barthel Index [20]) and three process measures of self-efficacy: General Self-Efficacy Scale (GSES) [21], Falls Self-Efficacy Scale – International (FES-I) [22,23] and Self-Efficacy for Exercise (SEE) [24]. The Barthel Index and GSES were collected at both baseline and follow-up, but FES-I and SEE were only completed at three month follow-up.

After the intervention was completed we carried out focus group interviews of patient and carer participants (Topic guide in Appendix 2). Separate focus groups were conducted for those in the control and intervention groups. Healthcare staff who delivered the intervention were also invited to separate focus groups at their nearest acute hospital site. Where staff were unable to attend, one-to-one telephone interviews were offered. Focus groups were recorded, transcribed and analysed thematically by two researchers. Patient

For peer review only

and carers were asked about their experience of rehabilitation in general and of taking part in the FEMuR study.

Patient and Public Involvement

Patient and public involvement representatives were involved in development of the original application for funding of the feasibility study and provided input on the choice of outcome measures, content of the intervention documents and patient facing materials. Topic guides for the focus groups were developed iteratively following feedback from early focus group participants. Participants who requested information on study findings were sent an overview of results and invited to input into the development of the future definitive study. The burden of the intervention to patients and their carers was discussed in the focus groups and formed an important part of assessing acceptability of the intervention.

Results

Recruitment of sites and rehabilitation teams

Rehabilitation team leads at three acute hospitals identified physiotherapists, based on the acute orthopaedic wards and occupational therapists and dual-trained technical instructors (TI) who were both acute and community based. The structure of the teams trained to deliver the intervention at each site differed depending on staff availability, with at least one Band 6 physiotherapist at each site who led the teams. As the physiotherapists in West and Central were based only within the acute hospital, the initial assessment session and introduction of the hip fracture workbook took place in the acute setting. In East, the physiotherapist was able to conduct this session with the patient in the community

For peer review only

following discharge. Rehabilitation teams were advised to support the patients in setting individual goals which could be worked on in the intervention sessions, with a particular focus on activities of daily living. Specific content of the session was decided at the discretion of the therapist and was dependent on individual patient need. Training sessions also included information on how to screen potential participants and how to complete intervention paperwork to capture detail on how additional sessions were used.

Response of rehabilitation teams

The initial recruitment period was planned to last six months. Due to staffing difficulties and the rurality of the West area, there were challenges delivering the intervention, and recruitment was slower than expected. Recruitment was extended for three months in Central and East, but was closed in the West.

Recruitment and reach in patient and carer participants

Rates of recruitment, eligibility and retention are given in Table 2. The main reasons for ineligibility were: lack of mental capacity 161 (49%), not living independently 61 (19%), younger than 65 years (13%), living out of area 30 (9%), treated without surgery 23 (7%). Patients were recruited after 193 (73%) eligible patients were approached with 176 (91%) of these agreeing to talk to the researcher. Those who were not approached had either been: discharged home before recruitment, died, lived in areas where it was not possible to deliver the intervention, were deemed by clinical staff to be too ill to take part, or there were safety concerns that would have prevented the intervention being delivered due to lone worker policies. The main reasons for lack of recruitment in those approached were: burdensome 60 (31%), or disliked the study or questionnaire 13 (7%). Information

For peer review only

concerning the number of visits it took to recruit participants were collected for 36 patients. The majority of patients had two visits, because recruitment occurred early in patients' recovery from surgery, and many requested a return visit to discuss the study after they had been discharged.

The retention rate was highest in the East and lowest in the West. The West encountered particular difficulties accessing staff for the trial, which might explain their poor retention rate. Nine patients withdrew from the study and four could not be contacted, so were lost to follow-up.

In addition, 41 carers were identified and 31 (75%) agreed to participate. Six carers withdrew from the study, and seven were lost to follow-up, leaving 18 (58%) who completed the follow-up questionnaire.

Intervention delivery

Data describing usual therapy care were only available from 35 participants recruited in the Central hospital, and associated community therapy teams, who were using Therapy Manager software. Five of these participants withdrew from the study and no further data regarding usual care were collected. Following discharge from the acute hospital, patients were discharged to their place of residence or for further rehabilitation in a community hospital prior to going home (Appendix 3). Ten patients had no details recorded relating to usual care following acute hospital discharge. Of the 20 patients who did have entries, four did not receive any face-to-face appointments with a healthcare professional, as their entries related to telephone calls to patients who were either uncontactable or declined further treatment. For the 16 patients who received an appointment, there was a median of

For peer review only

three appointments (n=4). The maximum number of appointments for one patient was 21. There was a total of 81 appointments for these 16 patients with 73 of these appointments (90%) conducted as home visits. Home visits were completed by different members of the therapy team (Appendix 4). If an assessment was required then a qualified physiotherapist (PT) or occupational therapist (OT) completed the visit, whilst subsequent visits following an agreed care plan were conducted by a technical instructor (TI). Most (90%) outpatient appointments were conducted by a physiotherapist (10% not recorded).

Activities in these usual rehabilitation sessions were categorised by the researcher as direct or indirect. Direct activities involved the practice of activities of daily living (25%), physical exercise (23%), phone calls with patients, discussion of progress and assessment of mood. Indirect activities were predominantly referrals to other services (33%), or contact with other members of the multidisciplinary team (30%).

Therapy Manager also recorded qualitative data. A number of patients were reluctant to engage in physical activity until they had been seen by a physiotherapist, even though in many cases they were told there would be a wait of at least four weeks.

Twenty-nine people were randomised to the enhanced rehabilitation intervention and details were available for 20 (reasons for missing data in Appendix 5). The majority (n=13) received all six sessions. The mean number of sessions delivered was 4.7 (standard deviation 1.6, range 1 to 6). One patient randomised to the intervention was discharged from the community hospital to a respite care home, so her intervention therapy sessions were delivered there.

For peer review only

TIs conducted the majority (55%) of intervention sessions, with 44% conducted by physiotherapists and the remaining 1% by more than one team member. The content of the intervention sessions depended upon individual patient need, at the discretion of the treating therapist. Therapists consistently completed the intervention paperwork detailing the types of activities and the time taken. Each session lasted approximately one hour, with an additional hour for travelling. In the intervention sessions, there was a lower rate of practising exercises (15%) and activities of daily living (14%) than usual care. Instead, there was more answering questions raised by the intervention workbook, working with goal setting diaries, giving feedback on progress and discussing emotional needs. For indirect activities, only 7% was used for discussion with the wider team and 4% for referring to other services. The remaining indirect activities included travel to appointments, writing notes, arranging further appointments and discussions with carers.

Delivery to individuals

Ten participants returned their goal-setting diaries and workbooks to the study team. All of the diaries had inputs from the therapists detailing the goals that were set in the initial assessment session. Five had also been updated by patients and their carers. These participants used the diaries extensively, updating their progress on the initial goals agreed and entered by the therapist and including new goals, which they had entered into the diary themselves. Three of these participants also completed quizzes and hip fracture story sections of the workbook. One of the workbooks was completed by a carer who described the challenges to the patient's recovery and what they were doing to overcome them.

Response of individual patients to the enhanced intervention or usual rehabilitation

For peer review only

Four focus groups were conducted with patients and carers and two with healthcare professionals involved in the intervention (Table 3). Due to the geographical spread of participants in the West, it was not possible to conduct a focus group in this area, although one participant from this area was able to attend a focus group in Central. Healthcare professionals delivering the intervention in the West were unable to attend focus groups, but one acute physiotherapist and three technical instructors participated in individual telephone interviews. Four themes emerged:

Theme 1 - Variation of usual care and its impact on delivering the intervention

The frequency and format of usual community rehabilitation varied because of tailoring to individual need, the availability of resources and the provision of support services such as re-ablement and falls prevention classes. One carer described this variation as a "*postcode lottery*" (male carer, control group). In the control group, the initial contact with therapists often needed to be initiated by the patient, relying on their self-motivation, which was not necessary for the intervention group.

According to therapists, there was large geographical variation in usual care ranging from multiple same day appointments to no rehabilitation whatsoever. This variation affected how the enhanced rehabilitation intervention was delivered. One therapist commented that when she delivered the intervention to patients with minimal usual care she would "*spread out the sessions, and then just pushed [the patient] harder, in the two weeks"* (physiotherapist). In contrast, where a comprehensive rehabilitation programme was available, she would deliver intervention sessions weekly in the confidence that at the end of the intervention period this provision would be continued through community run falls prevention or exercise schemes.

For peer review only

Therapists were concerned about supporting patients to set individualised goals when these might conflict with goals supported by other rehabilitation providers.

*"It was much harder when they had [another ongoing service], the re-ablement ones were much harder to actually, because somebody else was already setting what they were going to achieve" (*physiotherapist).

Theme 2 – The importance of goal-setting

Goal-setting was identified by therapists as playing an important role in engaging patients with their own recovery and in providing motivation to regain function and independence.

"I think [patients] probably more motivated because they can see the steps, to getting to that point. And why you are doing it" (physiotherapist).

The patient-held goal setting diary were appreciated by participants, as it gave them a direct focus and accountability for their goals.

*"You feel as if you have got a goal to get to, because you have put it in that book and you have got a goal." (*female patient, intervention group).

Therapists felt that the workbook and diary enabled patients to be more involved in their rehabilitation.

Theme 3 - The role of the therapist in providing reassurance about safe physical activities

The majority of patient participants reported anger and frustration when their physical ability to progress did not match their expectations, and they remained dependent on others.

For peer review only

“*Being incapacitated infuriated me so much”* (female patient, intervention group).

It was at this point that the physiotherapist played a pivotal role in managing expectations and reassuring patients that they were progressing normally. In the absence of this support there was a risk of patients losing motivation. This reassurance was important for giving patients the confidence to perform physical activities, as there was an underlying concern that they may otherwise do exercises which may be harmful.

“I had the security to know they were the right exercises, somebody there who gave them to me and you know they are qualified and they are telling you the right thing to do” (female patient, control group).

For participants in the control group, this lack of reassurance was a particular problem. Patients received a list of activities to avoid (hip precautions), but some were given no information about what exercises and activities were safe to perform, and wanted access to; “*somebody I could have just picked up the phone and said, how about this, should this be happening*” (female patient, control group).

Both groups identified this initial contact with therapists as vital for building their confidence and supporting their self-motivation for recovery.

“Once you have the information and the guidance on what to do, what not to do, I think we are intelligent enough to go away and do it, but it is just that initial guidance... we might be capable but you still need guidance” (male patient, control group).

Patients emphasised the importance of the intervention sessions in allowing them time to discuss their individual problems, particularly in the early stages of rehabilitation. This was facilitated by their relationship with the therapist or therapy team, where they felt

For peer review only

comfortable enough to ask questions without fear of being dismissed or considered a nuisance. This was in contrast with how they felt in the acute hospital, or in usual care, where they were less informed about the processes and unfamiliar with the staff. A good relationship with their therapist underpinned successful rehabilitation, and enabled them to engage in, and take responsibility for, their role within the recovery process.

"I felt as though it was a sort of team effort, and she [the therapist] was sort of team leader, and knew what to do, and then it is sort of from part of the team if you like." (male patient, intervention group).

Theme 4 - Acceptability of the extra therapy sessions

Patient feedback on the intervention workbook varied. Some patients appreciated the explanation of the mechanics of their fracture and their better understanding of the surgery used to fix it.

"I thought it was good because it did explain things, it did explain to you what happens with a fracture" (female patient, intervention group).

Other patients reflected on the comfort that this additional information gave them.

"I didn't know what to expect but I found I read [the workbook] profusely every day, and I did, I found it very, very helpful. It made me feel that I wasn't on my own" (female patient, intervention group).

Other participants found the workbooks less useful.

"I sort of read it once and thought well you know this isn't very useful" (male patient, intervention group).

For peer review only

Without exception, the most useful aspect of the intervention was the extra time that participants received with the therapy teams. The goal-setting diary and information workbook were seen as useful supporting documents to these extra sessions. Whilst therapists acknowledged the complex nature of delivering intervention sessions in an environment of varied usual care, it was generally accepted that the extra sessions were a great benefit to patients. The analysis of the focus groups also led to the development of the GUIDE tool (Figure 1) which summarises the role of the therapist in the rehabilitation process, incorporating important factors identified by patients and their carers.

Unintended consequences

There were nine adverse events, six were serious, two resulted in re-admission to the acute hospital and there was one death; none were related to participation in the study.

Testing the intervention theory

Correlations between the Barthel Index and the three process measures of self-efficacy were statistically significant and suggested that higher levels of activities of daily living were associated with higher scores of self-efficacy (Appendix 6). Similarly higher scores in the FES-I represent a greater fear of falling, which was associated with lower levels of activities of daily living. The strongest correlation was with the FES-I.

Discussion

Summary of main findings

For peer review only

This study took place in three sites across North Wales. Recruitment to the study was more difficult in the West because of its rurality and also staff shortages. The recruitment rate was highest in Central; the retention rate was highest in the East. Usual rehabilitation care was very variable with a median of three appointments; the enhanced rehabilitation group received a mean of five additional therapy sessions. Variation in usual care affected how the enhanced rehabilitation intervention was delivered. TIs carried out most of the sessions, which consisted of practising exercises and activities of daily living, goal-setting, answering questions raised by the workbook and giving feedback on progress. Goal-setting had an important role in engaging patients in their own recovery, which was assisted by the workbook and diary. All of the returned goal-setting diaries had inputs from the therapy team, and half had written comments by the patients and carers. Some participants did not find the workbook and diary useful, but all valued the extra therapy sessions. The physiotherapist was very important for managing patients' expectations and for reassuring them about what physical activity was safe to perform. The lack of reassurance was particularly problematic for some in the control group. There were statistically significant correlations between three process measures of self-efficacy and the Barthel index, which supported the theory underlying the intervention.

Strengths and weaknesses

This was a mixed-methods process evaluation performed concurrently with a randomised feasibility study which has examined recruitment, reach, dose delivered, intervention fidelity, unintended consequences, contextual factors and underlying theory. Rates of recruitment and retention were low, and this process evaluation informs trial methods, as

For peer review only

well as how to deliver the intervention for a future, definitive, phase III RCT. Although many commented that goal-setting was enhanced by the workbook and self-monitoring diaries, half of the collected workbooks were not completed, and some found them unhelpful. Feedback from participants and intervention delivery staff will result in further refinement of the workbook and diary and also the development of training materials before the definitive trial. Because this process evaluation was only part of a feasibility study it is not possible to comment on longer-term implementation of the enhanced rehabilitation intervention. The process data were analysed concurrently with the outcome data from the feasibility study, so the analysis of quantitative data was performed blind to treatment allocation; however, the qualitative findings and the feasibility study outcomes were discussed in relation to one another. It was not possible to collect data on usual rehabilitation from all participants in the intervention group, because the Therapy Manager software was only used by the rehabilitation teams in the Central area.

The enhanced rehabilitation intervention was delivered by physiotherapists and Tis, with very little input from OTs. This was due to the availability of physiotherapists and the shortage of occupational therapists in this health board during the study period. We believe that there is sufficient overlap in rehabilitation practice for these findings to be relevant to OTs as well. Also, the technical instructors who delivered most of the extra rehabilitation sessions worked with both physiotherapists and occupational therapists. The extra rehabilitation sessions concentrated on improving self-efficacy and personal goal-setting more than the practice of exercise and ADLs. We did manage to capture the practice of exercise and ADLs in participants' own time in a small number of participants who returned

For peer review only

their diaries, however we do not know how often participants in the control group practised their exercises and ADLs outside of therapy sessions.

Despite being a feasibility study with a small sample size, it was possible to gather some evidence supporting the underlying theory with regard to the importance of self-efficacy.

Comparison with previous literature

Qualitative interviews of participants in the Exercise-Plus RCT in the United States, of a motivational intervention designed to increase adherence to rehabilitation exercise also found that identifying goals and improving self-efficacy were important, and an exercise booklet provided useful visual cues [25]. A good relationship with the therapist providing individualised care and verbal encouragement resulted in participants reciprocating their therapists' kindness by completing the exercise programme. They also described constraints to exercise such as unpleasant sensations of pain and fatigue, lack of time and space, and discontinuing their exercise once baseline function was restored. A qualitative study of a rehabilitation programme in Taiwan found that when therapists emphasised social support and resilience, patients developed more self-confidence and independence [26]. A process evaluation of a rehabilitation intervention in Sweden found that hip fracture had long lasting 'social and existential' effects on patients necessitating both physical and emotional support during recovery [27]. The recent 'Hip Sprint'audit reviewed physiotherapy rehabilitation for hip fracture patients throughout the UK [28]. It found that usual rehabilitation care was variable with wide variation in the delay before home rehabilitation started, the amount and frequency of visits and the type of staff involved. Physiotherapists, and in particular physiotherapy assistants, provided most of the care.

Implications for a future definitive RCT

For peer review only

Several lessons have been learnt for delivering the enhanced rehabilitation intervention for a definitive phase III RCT (Box 1). Recruitment was harder in rural areas, especially in areas with staff shortages, which will be an important consideration when choosing sites for the definitive trial. Research staff need to remain flexible, be prepared to recruit after discharge home, keep visiting potential participants and possibly delay recruitment until later after surgery. Employing therapists directly by the research team or secondment from the research delivery workforce would avoid them being pulled back to clinical work during staffing shortages. The workbook and goal-setting diary need to be refined further in the light of feedback from patients, carers and clinicians. A mnemonic (GUIDE) for therapists has been developed following the qualitative research (Figure 1), which will be useful as a training tool for the therapy teams prior to a definitive phase III RCT. The collection of usual rehabilitation care data could be enhanced by using a patient-held treatment log completed by visiting therapists.

Acknowledgments

The FEMuR team would like to thank all participants who took part in the study. The authors would also like to thank Health and Care Research Wales for supporting participant recruitment, and the healthcare staff at BCUHB who identified potential participants and delivered the intervention.

Contributors

NHW was the chief investigator and grant holder, was responsible for study design, conduct and analysis and had overall responsibility for the study and acts as guarantor. JLR was the

For peer review only

study manager overseeing day-to-day conduct, participant recruitment, methodological input, conducted qualitative analysis for the focus groups. NUD was involved in participant recruitment, acquisition of quantitative and qualitative data and analysis. NT conducted the statistical analysis for the feasibility study. Her institution affiliation changed from Bangor University to the University of Sheffield during the study. VM was a co-investigator responsible for study design, provided health psychology expertise and methodological oversight throughout the study. MW was involved in the conduct of the study including maintenance of study documentation, acquisition of data, provided administrative support. AWP contributed to qualitative analysis of the focus groups. All authors were involved in drafting, revising and approving this manuscript.

Acknowledgements

We would like to thank our Patient and Public Representative Tricia Best for her invaluable input into the study.

Competing Interests

NHW, JLR, NUD, MW, NT, VM report a grant from NIHR HTA programme, for the conduct of the study. NHW reports additional grants from Public Health Wales, NIHR HTA and BCUHB, outside the submitted work.

Funding statement

This work was supported by the National Institute for Health Research's Health Technology Assessment Programme, grant number 11/33/03. The views and opinions expressed therein are those of the authors and do not necessarily reflect those of the HTA, NIHR, NHS or the Department of Health.

For peer review only

Ethics approval

The study received ethical approval from the UK NHS North Wales West Research Ethics Committee—West. Ref 13/WA/0402 and NHS Research and Development approval from the Betsi Cadwalader University Health Board (BCUHB) Internal Review Panel.

Availability of data and materials

The datasets used during the current study are available from the corresponding author on reasonable request.

References

1. Johansen A, et al., National Hip Fracture Database. National report 2013, C.E.a.E. Unit, Editor 013, Royal College of Physicians: London.

2. Bertram, M., et al., *Review of the long-term disability associated with hip fractures.* Injury Prevention, 2011. **17**(6): p. 365-370.

3. National Clinical Guideline Centre. Hip fracture: the management of hip fracture in adults | Guidance and guidelines | NICE. National Institute of Health and Care Excellence 2011. Accessed 2017. Available at http://www.nice.org.uk/guidance/cg124.

4. Handoll, H.H., et al., *Multidisciplinary rehabilitation for older people with hip fractures.* Cochrane Database of Systematic Reviews, 2009(4).

5. Handoll, H.H., C. Sherrington, and J.C. Mak, *Interventions for improving mobility after hip fracture surgery in adults.* Cochrane Database of Systematic Reviews, 2011(3).

6. Crotty, M., et al., *Rehabilitation interventions for improving physical and psychosocial functioning after hip fracture in older people.* Cochrane Database of Systematic Reviews,

For peer review only

2010(1).

7. Williams NH, Roberts JL, Din NU, Charles JM, Totton N, Williams M, Mawdesley K, Hawkes CA, Morrison V, Lemmey A, Edwards RhT, Hoare Z, Pritchard AW, Woods RT , Alexander S, Sackley C, Logan P, Wilkinson C, Rycroft-Malone J. A multidisciplinary rehabilitation package following hip fracture: Fracture in the Elderly Multidisciplinary Rehabilitation (FEMuR). Health Technol Assess 2017; 21 (44).

8. Medical Research Council. Developing and evaluating complex interventions: new guidance. London: Medical Research Council, 2008.

9. Roberts JL, Din NU, Williams M, Hawkes CA, Charles JM, Hoare Z, Morrison V, Alexander S, Lemmey A, Sackley C, Logan P, Wilkinson C, Rycroft-Malone J, Williams NH. Development of an evidence-based complex intervention for community rehabilitation of hip fracture patients using realist review, survey and focus group. BMJ Open 2017; 7: e014362.

10. Williams NH, Hawkes C, Din NU, Roberts J, Charles JM, Morrison V, Hoare Z, Edwards RhT, Andrew G, Alexander S, Lemmey A, Woods B, Sackley C, Logan P, Hunnisett D, Mawdesley K, Wilkinson C. Fracture in the Elderly Multidisciplinary Rehabilitation (FEMuR): study protocol for a Phase II randomised feasibility study of a multidisciplinary rehabilitation package following hip fracture [ISRCTN22464643] Pilot feasibility Studies 2015; 1: 13.

11. Williams NH, Roberts JL, Din NU, Totton N, Charles JM, Hawkes CA, Morrison V, Hoare Z, Williams M, Pritchard AW, Mawdesley K, Hawkes CA, Morrison V, Lemmey A, Edwards RhT, Hoare Z, Pritchard AW, Alexander S, Lemmey A, Woods RT, Sackley C, Logan P, Edwards RhT, Wilkinson C. Fracture in the Elderly Multidisciplinary Rehabilitation (FEMuR): a phase II randomised feasibility study of a multidisciplinary rehabilitation package following hip fracture. BMJ Open 2016; 6: e012422.

For peer review only

12. Hodkinson HM. Evaluation of a mental test score for assessment of mental impairment in the elderly. Age Ageing 1972; 1: 233–238.

13. Steckler A, Linnan L (Eds.) Process evaluation for public health interventions and research. San Francisco: Jossey-Bass, 2002.

14. Moore G, Audrey S, Barker M, Bond L, Bonell C, Hardeman W, Moore L, O'Cathain A, Tinati T, Wight D, Baird J. Process evaluation of complex interventions: UK Medical Research Council (MRC) Guidance. MRC Population Health Science Research Network, 2014.

15. Mastersson-Allgar P. Advancing process evaluation research within the field of Neurological rehabilitation. PhD thesis, Bangor University, 2016.

16. Grant A, Treweek S, Dreischulte T, Foy R, Guthrie B. Process evaluations for cluster randomised trials of complex interventions: a proposed framework for designing and reporting. Trials 2013; 14: 15.

17. Oakley A, Strange V, Bonell C, Allen E, Stephenson J, RIPPLE Study Team. Process evaluation in randomised controlled trials of complex interventions. BMJ 2006; 332: 413-416.

18. Moore GF, Raisanen L, Moore L, Din NU,Murphy S. Mixed-method process evaluation of the Welsh National Exercise Referral Scheme. Health Education 2013; 113(6): 476-501.

19. Pawson R, Tilley N. Realistic evaluation, London: Sage, 1997.

20. Mahoney F, Barthel D. Functional evaluation: the Barthel index. Maryland State Med J. 1965;14:56–61.

21. Schwarzer R, Jerusalem M. Generalized self-efficacy scale. In: Weinman J, Wright S, Johnston M, editors. Measures in health psychology: a user's portfolio causal and control beliefs. Windsor, UK: NFER-NELSON; 1995. p. 35–7.

For peer review only

22. Hauer K, Yardley L, Beyer N, Kempen G, Dias N, Campbell M, et al. Validation of the Falls Efficacy Scale and Falls Efficacy Scale International in geriatric patients with and without cognitive impairment: results of self-report and interview-based questionnaires. Gerontology. 2010;56:190–9.

23. Yardley L, Beyer N, Hauer K, Kempen G, Piot-Ziegler C, Todd C. Development and initial validation of the Falls Efficacy Scale-International (FES-I). Age Ageing. 2005;34:614–9.

24. Resnick B, Jenkins LS. Testing the reliability and validity of the self-efficacy for exercise scale. Nurs Res. 2000;49:154–9.

25. Resnick B, Orwig D, Wehren L, Zimmerman S, Simpson M, Magaziner J. The Exercise Plus program for older women post hip fracture: participant perspectives. Gerontologist. 2005;45:539–44.

26. Huang T, Acton G. Ways to maintain independence among Taiwanese elderly adults with hip fractures: A qualitative Study. Geriatric Nurs. 2009;30:28-35

27. Ziden L, Wenestam C, Hansson-Scherman M. A life-breaking event: early experiences of the consequences of a hip fracture for elderly people. Clin Rehab. 2008;22:801–11.

28. Royal College of Physicians. Recovering after a hip fracture: helping people understand physiotherapy in the NHS. Physiotherapy 'Hip Sprint' audit report 2017. London: RCP, 2018.

For peer review only

Table 1 Process evaluation questions and methods for evaluating

Component	Process evaluation questions	Research methods	Stage of study to collect data
Recruitment of sites and rehabilitation teams	How are sites and teams recruited?	Documentation of recruitment process by research team	Pre-intervention
	Which sites and teams agree to participate?	Quantitative comparison of recruited and non-recruited sites	
Response of rehabilitation teams	How is the enhanced intervention adopted by the rehabilitation teams	Quantitative examination of case report forms and qualitative interviews of rehabilitation team members	During and following the intervention
Recruitment and reach in patient and carer participants	How many are recruited into the feasibility study? Are they representative?	Quantitative comparison between feasibility study and anonymised cohort	During the intervention
	Who is recruited into the feasibility study? What are the reasons for non-recruitment?	Examination of recruitment log	During the intervention
Intervention delivery	What rehabilitation intervention is delivered? Is it what was intended by the researchers?	Quantitative examination of case report forms and of electronic data entered onto Therapy Manager software	During the intervention
Delivery to individuals	What intervention is delivered to each participant?	Quantitative examination of case report forms and of electronic data entered onto Therapy Manager software	During the intervention
	Is the delivered intervention the one intended by the researchers?	Measurement of intervention fidelity: completion of workbook tasks, completion of diaries, number and content of therapy sessions	During the intervention
Response of individual patients to the enhanced intervention or usual rehabilitation	How do the patient participants respond?	Qualitative analysis of focus group data about patient participants' experience and response to the intervention and to usual care	Following the intervention
Response of carer participants	Effects on carers	Qualitative analysis of focus group data about carers' experiences	Following the intervention
Unintended consequences	Are there unintended changes in processes and outcomes related to the intervention and unrelated to care?	Quantitative examination of adverse effects reports, health service activity data from patient completed questionnaires and routinely collected electronic sources. Qualitative analysis of focus group data from patients and	During and following the intervention

For peer review only

		their carers	
Theory	What theory has been used to develop the intervention?	Quantitative data analysis of process outcome measures to assess predicted relationships	Following the intervention
Context	What is the wider context in which the feasibility study is conducted?	Realist review of the rehabilitation literature, survey of current rehabilitation practice, focus groups of patients, carers and rehabilitation professionals. Quantitative comparison with anonymised cohort	Pre-intervention (phase I study) and during the intervention

For peer review only

Table 2 **Eligibility, recruitment and retention rates according to acute hospital site**

Number of Patients	West	Central	East	Total
**Screened (rate)**	147	235	211	593
**Eligible (rate)**	75 (51%)	103 (44%)	88 (42%)	266 (45%)
**Recruited (rate)**	11 (15%)	35 (34%)	16 (18%)	62 (23%)
**Retained (rate)**	4 (36%)	29 (83%)	16 (100%)	49 (79%)

For peer review only

Table 3 Focus group participants' characteristics

Participant type	Location	Attendees
Patient and carers in control group	East	2 female patients, 1 male patient, 2 male carers (n=5)
Patient and carers in control group	Central	2 female patients, 1 male patient, 1 female carer (n=4)
Patient and carers in intervention group	East	3 female patients (n=3)
Patient and carers in intervention group	Central	2 male patients, 2 female patients, 1 male carer, 2 female carers (n=7)
Healthcare professionals	East	Clinical specialist physiotherapist, 2 orthopaedic physiotherapists, physiotherapy technical instructor (n=4)
Healthcare professionals	Central	Orthopaedic acute physiotherapist, rotational physiotherapist, physiotherapy technical instructor (n=3)

For peer review only

Box 1 Ten lessons learnt for a future definitive randomised controlled trial

Sites	1	Consider staff availability and rurality when recruiting sites
Therapy staff	2	Consider employing therapy staff directly or second from research delivery teams
Participant recruitment	3	Recruitment flexibility – including after discharge home
	4	Keep visiting potential participants
	5	Delay recruitment until later after surgery
Trial-specific training	6	Use the mnemonic GUIDE as a training aid
	7	Stress the importance of the first home visit to reassure participants about safe activities
	8	Value the emotional support provided by the technical instructors
Usual care recording	9	Patient-held treatment log completed by visiting therapists.
Workbook	10	Further refine the workbook in the light of feedback

For peer review only

Figure Legends

Figure 1 GUIDE mnemonic for therapists involved in rehabilitation following hip fracture

For peer review only

For peer review only

For peer review only

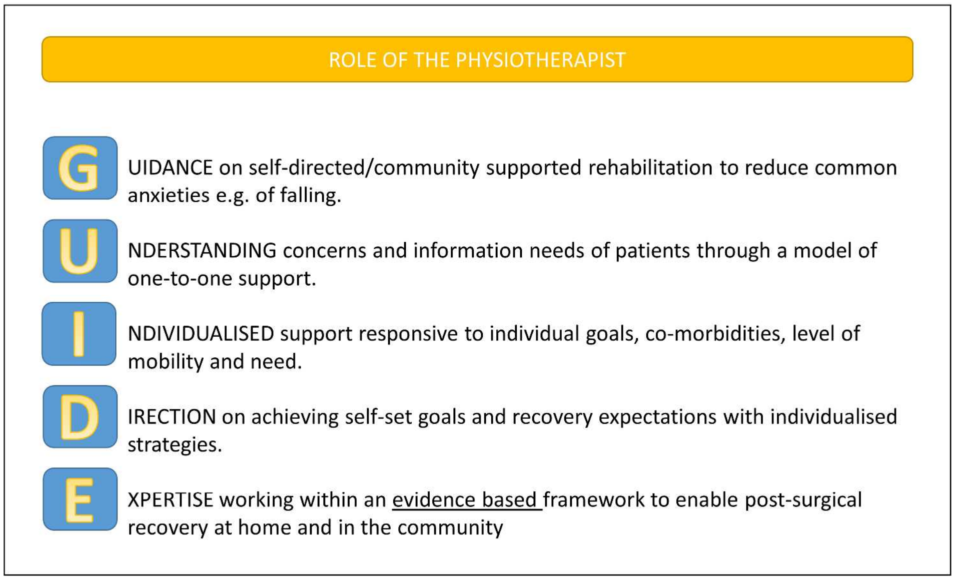

Figure 1. GUIDE mnemonic for therapists involved in rehabilitation following hip fracture

275x190mm (300 x 300 DPI)

Appendix 1 Case report form

Session number:

Patient Study ID: ☐☐☐☐☐

Date: ____ /____ /________ **Total amount of time used for this session:**

Role(s) of person/people conducting visit: Occupational therapist / Physiotherapist / OT assistant / Physiotherapy assistant / Technical instructor / Other

Location of session: Patient's own home / Care home where patient lives permanently / Community hospital outpatients / Acute hospital outpatients / ______________________

Other

1. Please indicate what ***indirect*** care activities you carried out for the patient during this session, and for how long:

Activity	Time (e.g. 10 minutes)	Further details
Referring to another service		
Phone contact with patient		
Contact with MDT about patient		
Patient admin		
Travel to and from session		
Other, please specify.		

2. Please indicate what ***direct*** care activities you carried out for the patient during this session, and for how long:

Activity	Time (e.g. 20 minutes)	Further details
Assessment/re-assessment		
Physical exercises		
Activities of daily living practice		
Workbook		
Discussion of care and progress		

For peer review only

Activity	Time (e.g. 20 minutes)	Further details
Discussion of referral to a follow on service e.g. social groups, falls groups, outpatients physio		
Travel to and from session		
Other, please specify		

3. If you spent time on the ***workbook*** in the session, please indicate what this involved:

Activity	Time (e.g. 5 minutes)	Further details
Goal setting		
Reviewing progress		
Answering patient questions stimulated by workbook		
Emotional support		
Other, please specify		

For peer review only

Appendix 2 Focus group topic guides

Patient and carer topic guide – control group

- What was your experience of the rehabilitation you received after your hip fracture, specifically regarding physiotherapy and exercise?
- What went well, what could be improved? Having experienced fracture and the rehabilitation involved, what would you recommend to improve care?
- If there are any attending the focus group, what were the experiences of participants who were discharged to nursing/residential care, or those who were more dependent on carers?

- Did your therapists give you exercises to do?
  - How confident did you feel about practising them?
  - Did your confidence change over time? What changed this?
- Were you given any written information to keep with you?
  - How helpful was this? Was there any other information you would have found useful?
- How involved did you feel in planning your recovery and rehabilitation?
- Often people say they were afraid of falling again after having a hip fracture, did you experience this?
  - Was there anything in your rehabilitation that helped reduce the fear of falling?
  - Did you attend a falls prevention class? How did you find out about this and was it useful?
- How did you feel about being approached to take part in the study?
  - Did you understand the information you were given?

For peer review only

- What made you decide to take part?
- How did you feel about the questions you were asked in the hospital and at the follow up, especially about how long they took and how well you were able to understand the questions and their relevance?

- How did you feel about not knowing which study group you would be in when you agreed to take part?
    - How would you have felt if you had been in the other group?
- Did you do some exercise tests with your community physiotherapists at the end of the study?
    - How did you feel about being asked to do these?
- Has anything changed for you as a consequence of being in the study?
    - If you were asked to take part now, would you make the same decision?

Patient and carer topic guide – intervention group

- What was your experience of the rehabilitation you received after your hip fracture?
    - What went well, what could be improved?
    - If there are any attending the focus group, what were the experiences of participants who were discharged to nursing/residential care, or those who were more dependent on carers?
- TASK - Rank the following aspects of the intervention according to how useful they were:
    - Extra time with a therapist
    - Information in the workbook
    - Being involved in setting your own goals

For peer review only

- Keeping a diary to look at progress
- Discussion of ranking
- Did you feel confident to suggest goals to your therapists and how did that help you?
- What did you like best about the workbook?
    - Was there anything you felt the workbook was missing?
- Were you aware of the extra sessions which you received because you were in the study?
    - Were these sessions used differently to the care as usual sessions?
    - Did you notice any improvements in your recovery as a result of this extra time?
- Did your therapists give you exercises to do?
    - How confident did you feel about practising them?
    - Did your confidence change over time? What changed this?
- Often people say they were afraid of falling again after having a hip fracture, did you experience this?
    - Was there anything in your rehabilitation that helped reduce the fear of falling?
    - Did you attend a falls prevention class? How did you find out about this and was it useful?
- How did you feel about being approached to take part in the study?
    - Did you understand the information you were given?
    - What made you decide to take part?
    - How did you feel about the questions you were asked in the hospital and at the follow up, especially about how long they took and how well you were able to understand the questions and their relevance?

For peer review only

- Did you do some exercise tests with your community physiotherapists at the end of the study?
    - How did you feel about being asked to do these?
- How did you feel about not knowing which study group you would be in when you agreed to take part?
    - How would you have felt if you had been in the other group?
- Has anything changed for you as a consequence of being in the study?
    - If you were asked to take part now, would you make the same decision?

Healthcare professional topic guide

- Experiences of the enhanced rehabilitation intervention
    - What went well?
    - What could be improved?
- Experiences of extra therapist time
- Awareness of extra time available to patients in the study
    - How did if work, what went well what needs to be improved
    - How did you use the extra time with patients? Do you think it made a difference?
- Views of the Workbook
    - Did you use it?
    - If so how, what was useful, what could be improved, was anything missing you think would have been helpful?

For peer review only

- Do you feel the intervention made any difference to the way patients engaged with you and participated in their rehabilitation?
    - If so how/if not why?
    - What was your experience the goal setting and feedback in the work book? Similar/different to usual way you work/good points/areas for improvement
    - What was your experience of the information sections? Useful to you/useful to patients/anything you weren't aware of before/ anything that was missing?
- Experiences of working with patients with cognitive impairments and their carers
    - How did the intervention go?
- Experiences of working with patients who were discharged to long term residential/nursing care
    - How did the intervention go?
- Feasibility of physiotherapists conducting the physical measures at 3 month follow-up
- Acceptability of the outcome measures for patients
    - Timing; Number; Content
- Anything else we haven't talked about you think has been important in working with this intervention?

For peer review only

Appendix 3 Rehabilitation pathways in usual care on discharge from the central acute hospital following surgical repair of hip fracture

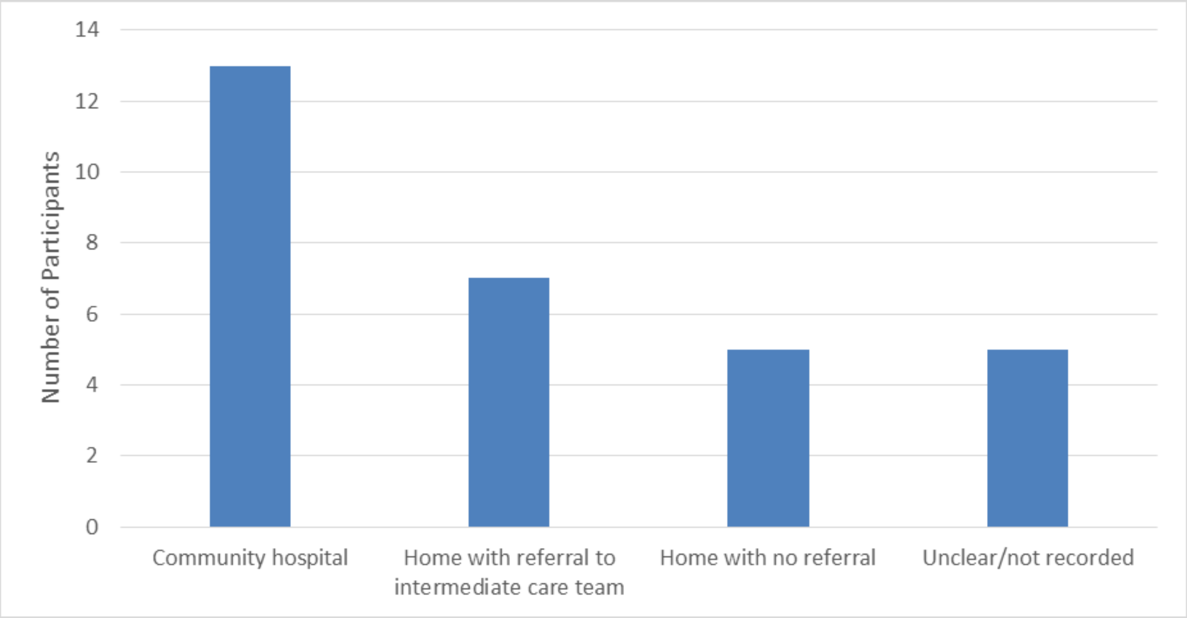

review only

Appendix 4 Percentage of home visits conducted by different members of the rehabilitation team for usual care

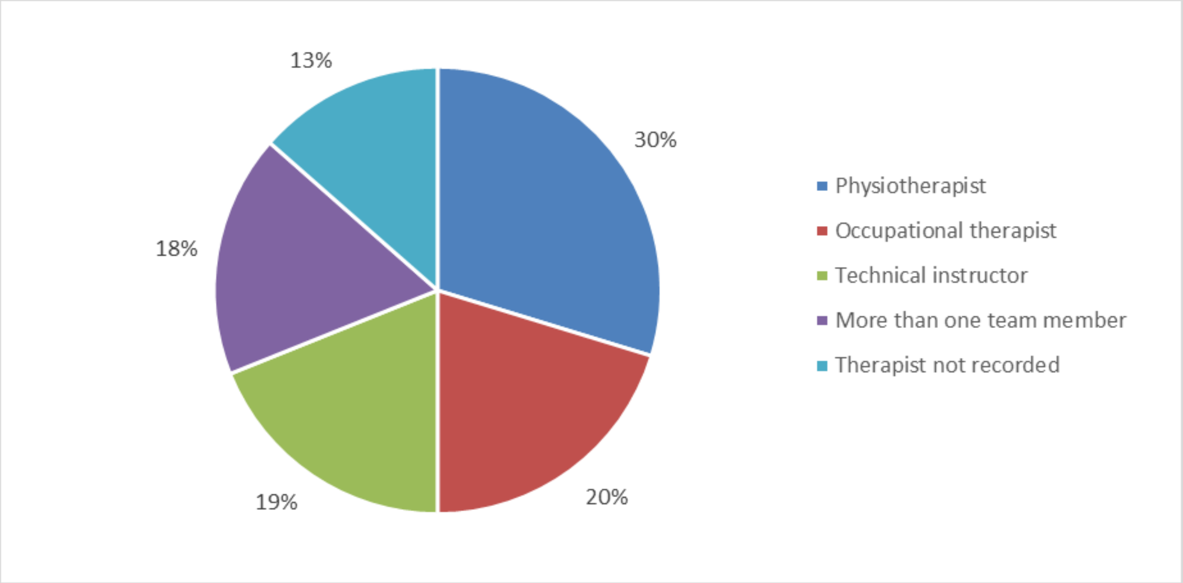

For peer review only

Appendix 5 Reasons for missing data on intervention session use

Reason	No. of participants	Area
Withdrawn	4	Gwynedd, Anglesey, Conwy and Denbighshire
Area not covered by therapist	1	Gwynedd and Anglesey
Declined intervention sessions	1	Conwy and Denbighshire
Could not be contacted to arrange sessions	1	Conwy and Denbighshire
Received intervention but therapist not returned paperwork	1	Gwynedd and Anglesey
Therapist delivering sessions moved to different area and could not complete intervention	1	Gwynedd and Anglesey

For peer review only

For peer review only

Appendix 6 Correlation analysis of Barthel Index with measures of self-efficacy

Self-Efficacy Measures	Barthel Index at baseline<br>Correlation (95% Confidence Intervals)	Barthel Index at 3 months<br>Correlation (95% Confidence Intervals)
General Self-Efficacy Scale (Baseline and 3 Months)	r_{55}= 0.43 (0.12 to 0.68), p=0.001	r_{37}= 0.59 (0.23 to 0.82), p<0.001
Falls Efficacy Scale – International* (3 Months)	Not Collected	r_{34}= -0.68 (-0.83 to -0.45), p<0.001
Self-Efficacy for Exercise Scale (3 Months)	Not Collected	r_{33}= 0.62 (0.26 to 0.82), P<0.001

* High values represent high levels of fear of falling

For peer review only

BMJ Open

Mixed methods process evaluation of an enhanced community-based rehabilitation intervention for elderly hip fracture patients.

Journal:	*BMJ Open*
Manuscript ID	bmjopen-2018-021486.R2
Article Type:	Research
Date Submitted by the Author:	15-Jun-2018

Complete List of Authors:	Roberts, Jessica; Bangor University, North Wales Centre for Primary Care Research<br>Pritchard, Aaron; Betsi Cadwaladr University Health Board, Research and Development<br>Williams, Michelle; Bangor University<br>Totton, Nikki; Bangor University College of Health and Behavioural Sciences, North Wales Organisation for Randomised Trials in Health; University of Sheffield, Clinical Trials Research Unit<br>Morrison, Val; School of Psychology, Bangor University<br>Din, Nafees; Bangor University, North Wales Centre for Primary Care Research<br>Williams, Nefyn; University of Liverpool, Department of Health Services Research

**Primary Subject Heading**:	Rehabilitation medicine
Secondary Subject Heading:	Health services research, Qualitative research, Research methods, Geriatric medicine
Keywords:	Process evaluation, Feasibility study, Hip fracture, Proximal femoral fracture, REHABILITATION MEDICINE, Self-efficacy

SCHOLARONE™
Manuscripts

Mixed methods process evaluation of an enhanced community-based rehabilitation intervention for elderly hip fracture patients.

Jessica L Roberts PhD[1], Aaron W Pritchard MSc[2], Michelle Williams BSc[1], Nikki Totton PhD[3], Val Morrison PhD CPsychol[4], Nafees Ud Din MSc MFDS RCS[1], Nefyn H Williams PhD FRCGP[5].

[1]School of Healthcare Sciences, Bangor University, UK

[2]Betsi Cadwaladr University Health Board, North Wales, UK

[3]School of Health and Related Research, Sheffield University, UK

[4]School of Psychology, Bangor University, UK

[5]Department of Health Services Research, University of Liverpool, UK

Corresponding author: Dr Jessica L Roberts, North Wales Centre for Primary Care Research, School of Healthcare Sciences, Bangor University, Cambrian 2, Wrexham Technology Park, Wrexham LL13 7YP, UK.

Tel: +44 (0)1248 383516 e-mail: j.l.roberts@bangor.ac.uk

For peer review only

Abstract

Objectives: To describe the implementation of an enhanced rehabilitation programme for elderly hip fracture patients with mental capacity, in a randomised feasibility study compared with usual rehabilitation. To compare processes between the two, and to collect the views of patients, carers and therapy staff about trial participation.

Design: Mixed methods process evaluation in a randomised feasibility study

Setting: Patient participants were recruited on orthopaedic and rehabilitation wards; the intervention was delivered in the community following hospital discharge.

Participants: Sixty one older adults (aged ≥65) recovering from surgical treatment (replacement arthroplasty or internal fixation) following hip fracture, who were living independently prior to fracture and had mental capacity; and 31 of their carers.

Interventions: Usual care (control) or usual care plus an enhanced rehabilitation package (intervention). The enhanced rehabilitation consisted of a patient-held information workbook, goal-setting diary and up to six additional therapy sessions.

Process evaluation components: Recruitment of sites and rehabilitation teams, response of rehabilitation teams, recruitment and reach in patient and carer participants, intervention delivery, delivery to individuals, response of individual patients to the enhanced intervention or usual rehabilitation, response of carer participants, unintended consequences, testing intervention theory and context.

Results: Usual rehabilitation care was very variable. The enhanced rehabilitation group received a mean of five additional therapy sessions. All of the returned goal-setting diaries had inputs from the therapy team, and half had written comments by the patients and

For peer review only

carers. Focus group themes: variation of usual care and its impact on delivering the intervention; the importance of goal setting; the role of the therapist in providing reassurance about safe physical activities; acceptability of the extra therapy sessions.

Conclusions: Lessons learnt for a future definitive RCT include how to enhance recruitment, and improve training materials, the workbook, delivery of the extra therapy sessions and recording of usual rehabilitation care.

Trial registration: ISRCTN22464643 Registered 21 July 2014

Key words: Process evaluation, feasibility study, hip fracture, proximal femoral fracture, rehabilitation medicine, self-efficacy

Strengths and limitations of this study

- Mixed-methods process evaluation of a phase II randomised feasibility study which has examined recruitment, reach, dose delivered, intervention fidelity, unintended consequences, contextual factors and underlying theory.
- It will inform the delivery of a future, definitive, phase III RCT.
- It is not possible to comment on the longer-term implementation of the enhanced rehabilitation intervention, because this process evaluation was of a feasibility study with only a three month follow-up.
- The participants did not include people with severe cognitive impairment, as the lack of mental capacity was an exclusion criterion.

For peer review only

- Despite being a feasibility study with a small sample size, it was possible to gather some evidence supporting the underlying theory with regard to the importance of self-efficacy.

For peer review only

Background

Proximal femoral fracture, known as hip fracture, is a major health problem in the elderly [1], associated with a reduced ability to conduct activities of daily living independently [2]. Guidelines from the National Institute of Health and Care Excellence recommend the use of multidisciplinary rehabilitation programmes to maximise patient's recovery potential [3], but there is insufficient evidence of effectiveness and cost-effectiveness and the importance of individual components of these programmes in achieving desirable outcomes is poorly understood [4-6].

Study context

A study funded by the HTA programme [7] completed the first two phases of the MRC framework for complex interventions [8]. The first phase developed an enhanced rehabilitation intervention from the following working theory [9]:

"In the context of patients with a great range and variety of pre-fracture physical and mental comorbidities affecting their ability to meet rehabilitation goals, a tailored intervention incorporating increased amount of high quality practice of exercise and activities of daily living leads to better confidence, mood, function, mobility and reduced fear of falling."

In addition to usual care, the intervention included:

- Six home-based therapy sessions delivered by physiotherapists or occupational therapists with the assistance of technical instructor providing reliable and consistent care.

For peer review only

- A novel, patient-held, workbook containing information on hip fracture, what to expect from rehabilitation, information about their role in their recovery, importance of physical activity and maintaining functional activities, and signposting to other services. The workbook contained a page of questions and Likert scale type response options to encourage participants to provide feedback on their workbook.
- A diary to facilitate patient-led goal setting, promote engagement and increase self-management.

A logic model described how the intervention components related to the programme theory [10].

The second phase of the study was a randomised feasibility study, which assessed the acceptability of the new rehabilitation programme and the feasibility of trial methods [10, 11]. Participants in the feasibility study were recruited from three acute hospitals across North Wales: East, Central and West. The rehabilitation intervention was delivered in the community. Participants were adults aged 65 years or older who had received surgical treatment for hip fracture, had been living independently prior to the hip fracture, had mental capacity as assessed by their clinical team, and received rehabilitation in the North Wales area. Between June 2014 and March 2015, 61 participants were randomised to usual care (control) or usual care plus the enhanced rehabilitation package (intervention). The mean Abbreviated Mental Test Score for these participants was 9.1 (standard deviation 1.3 and range 5 to 10). A score of 8 or less suggests cognitive impairment [12].

In addition, participants recruited to the feasibility study were compared with an anonymised cohort of all patients admitted to the same acute hospitals with a proximal femoral fracture over a similar time period. Compared with this cohort, the study

For peer review only

participants were younger, less likely to be re-admitted to hospital and less likely to die. Outcomes were measured at baseline and at three months' follow-up and included: disability, activities of daily living, anxiety and depression, health utility, health service resource use, hip pain intensity, self-efficacy, fear of falling, physical function and carer strain.

Guidance from the UK Medical Research Council for developing and evaluating complex interventions recommends conducting a process evaluation, in order to 'explain discrepancies between expected and observed outcomes, and to provide insights to aid implementation' [8]. The aims of this process evaluation were to describe the implementation of an enhanced rehabilitation programme for elderly hip fracture patients with mental capacity. The specific objectives were to:

- Describe the implementation of the enhanced rehabilitation programme in the intervention group and usual rehabilitation in the control group;
- Describe and compare processes between the two forms of rehabilitation;
- Collect data from trial participants (patients, carers and therapy staff) about their experience of taking part in the trial;
- Collect data about contextual factors and test the theory underlying the intervention.

Methods

The study was influenced by Steckler and Linnan's process evaluation framework [13], and other proposed frameworks for designing and reporting process evaluations [14-16], other

For peer review only

process evaluations of trials of complex interventions [17,18] as well as realist evaluation [19] (Table 1).

Mixed methods were used to collect process data. Routinely collected electronic health records using Therapy Manager software were used to extract usual rehabilitation activity data for participants in both intervention and control groups. The content of the additional enhanced rehabilitation sessions were recorded by therapy staff onto case report forms. These described how the sessions were used for each patient, including the length of the session, where the session was delivered and the type of activities undertaken (Appendix 1). Workbooks and goal-setting diaries were collected from patients at follow-up and examined for degree of completion. Questionnaires completed by patients and carers contained health service resource use data. We used descriptive statistics to compare recruitment rates between the sites, and to describe the rehabilitation components used from the case report forms, routinely collected electronic records, and the completed workbooks and diaries.

We tested the theory underlying the intervention by testing the correlation between the main outcome measure (Barthel Index [20]) and three process measures of self-efficacy: General Self-Efficacy Scale (GSES) [21], Falls Self-Efficacy Scale – International (FES-I) [22,23] and Self-Efficacy for Exercise (SEE) [24]. The Barthel Index and GSES were collected at both baseline and follow-up, but FES-I and SEE were only completed at three month follow-up.

After the intervention was completed we carried out focus group interviews of patient and carer participants (Topic guide in Appendix 2). Separate focus groups were conducted for those in the control and intervention groups. Healthcare staff who delivered the

For peer review only

intervention were also invited to separate focus groups at their nearest acute hospital site. Where staff were unable to attend, one-to-one telephone interviews were offered. Focus groups were recorded, transcribed and analysed thematically by two researchers. Patient and carers were asked about their experience of rehabilitation in general and of taking part in the FEMuR study.

Patient and Public Involvement

Patient and public involvement representatives were involved in the development of the original application for funding of the feasibility study and provided input on the choice of outcome measures, content of the intervention documents and patient facing materials. Topic guides for the focus groups were developed iteratively following feedback from early focus group participants. Participants who requested information on study findings were sent an overview of the results and invited to input into the development of the future definitive trial. The burden of the intervention to patients and their carers was discussed in the focus groups and formed an important part of assessing acceptability of the intervention.

Results

Recruitment of sites and rehabilitation teams

Rehabilitation team leads at three acute hospitals identified physiotherapists, based on the acute orthopaedic wards and occupational therapists and dual-trained technical instructors (TI) who were both acute and community based. The structure of the teams trained to deliver the intervention at each site differed depending on staff availability, with at least one Band 6 physiotherapist at each site who led the teams. As the physiotherapists in West

For peer review only

and Central were based only within the acute hospital, the initial assessment session and introduction of the hip fracture workbook took place in the acute setting. In East, the physiotherapist was able to conduct this session with the patient in the community following discharge. Rehabilitation teams were advised to support the patients in setting individual goals which could be worked on in the intervention sessions, with a particular focus on activities of daily living. Specific content of the session was decided at the discretion of the therapist and was dependent on individual patient need. Training sessions also included information on how to screen potential participants and how to complete intervention paperwork to capture detail on how additional sessions were used.

Response of rehabilitation teams

The initial recruitment period was planned to last six months. Due to staffing difficulties and the rurality of the West area, there were challenges delivering the intervention, and recruitment was slower than expected. Recruitment was extended for three months in Central and East, but was closed in the West.

Recruitment and reach in patient and carer participants

Rates of recruitment, eligibility and retention are given in Table 2. The main reasons for ineligibility were: lack of mental capacity 161 (49%), not living independently 61 (19%), younger than 65 years (13%), living out of area 30 (9%), treated without surgery 23 (7%). Patients were recruited after 193 (73%) eligible patients were approached with 176 (91%) of these agreeing to talk to the researcher. Those who were not approached had either been: discharged home before recruitment, died, lived in areas where it was not possible to deliver the intervention, were deemed by clinical staff to be too ill to take part, or there

For peer review only

were safety concerns that would have prevented the intervention being delivered due to lone worker policies. The main reasons for lack of recruitment in those approached were: burdensome 60 (31%), or disliked the study or questionnaire 13 (7%). Information concerning the number of visits it took to recruit participants were collected for 36 patients. The majority of patients had two visits, because recruitment occurred early in patients' recovery from surgery, and many requested a return visit to discuss the study after they had been discharged.

The retention rate was highest in the East and lowest in the West. The West encountered particular difficulties accessing staff for the trial, which might explain their poor retention rate. Nine patients withdrew from the study and four could not be contacted, so were lost to follow-up.

In addition, 41 carers were identified and 31 (75%) agreed to participate. Six carers withdrew from the study, and seven were lost to follow-up, leaving 18 (58%) who completed the follow-up questionnaire.

Intervention delivery

Data describing usual therapy care were only available from 35 participants recruited in the Central hospital, and associated community therapy teams, who were using Therapy Manager software. Five of these participants withdrew from the study and no further data regarding usual care were collected. Following discharge from the acute hospital, patients were discharged to their place of residence or for further rehabilitation in a community hospital prior to going home (Appendix 3). Ten patients had no details recorded relating to usual care following acute hospital discharge. Of the 20 patients who did have entries, four

For peer review only

did not receive any face-to-face appointments with a healthcare professional, as their entries related to telephone calls to patients who were either uncontactable or declined further treatment. For the 16 patients who received an appointment, there was a median of three appointments (n=4). The maximum number of appointments for one patient was 21. There was a total of 81 appointments for these 16 patients with 73 of these appointments (90%) conducted as home visits. Home visits were completed by different members of the therapy team (Appendix 4). If an assessment was required then a qualified physiotherapist (PT) or occupational therapist (OT) completed the visit, whilst subsequent visits following an agreed care plan were conducted by a technical instructor (TI). Most (90%) outpatient appointments were conducted by a physiotherapist (10% not recorded).

Activities in these usual rehabilitation sessions were categorised by the researcher as direct or indirect. Direct activities involved the practice of activities of daily living (25%), physical exercise (23%), phone calls with patients, discussion of progress and assessment of mood. Indirect activities were predominantly referrals to other services (33%), or contact with other members of the multidisciplinary team (30%).

Therapy Manager also recorded qualitative data. A number of patients were reluctant to engage in physical activity until they had been seen by a physiotherapist, even though in many cases they were told there would be a wait of at least four weeks.

Twenty-nine people were randomised to the enhanced rehabilitation intervention and details were available for 20 (reasons for missing data in Appendix 5). The majority (n=13) received all six sessions. The mean number of sessions delivered was 4.7 (standard deviation 1.6, range 1 to 6). One patient randomised to the intervention was discharged from the

For peer review only

community hospital to a respite care home, so her intervention therapy sessions were delivered there.

TIs conducted the majority (55%) of intervention sessions, with 44% conducted by physiotherapists and the remaining 1% by more than one team member. The content of the intervention sessions depended upon individual patient need, at the discretion of the treating therapist. Therapists consistently completed the intervention paperwork detailing the types of activities and the time taken. Each session lasted approximately one hour, with an additional hour for travelling. In the intervention sessions, there was a lower rate of practising exercises (15%) and activities of daily living (14%) than usual care. Instead, there was more answering questions raised by the intervention workbook, working with goal setting diaries, giving feedback on progress and discussing emotional needs. For indirect activities, only 7% was used for discussion with the wider team and 4% for referring to other services. The remaining indirect activities included travel to appointments, writing notes, arranging further appointments and discussions with carers.

Delivery to individuals

Ten participants returned their goal-setting diaries and workbooks to the study team. All of the diaries had inputs from the therapists detailing the goals that were set in the initial assessment session. Five had also been updated by patients and their carers. These participants used the diaries extensively, updating their progress on the initial goals agreed and entered by the therapist and including new goals, which they had entered into the diary themselves. Three of these participants also completed quizzes and hip fracture story sections of the workbook. One of the workbooks was completed by a carer who described the challenges to the patient's recovery and what they were doing to overcome them.

For peer review only

Response of individual patients to the enhanced intervention or usual rehabilitation

Four focus groups were conducted with patients and carers and two with healthcare professionals involved in the intervention (Table 3). Due to the geographical spread of participants in the West, it was not possible to conduct a focus group in this area, although one participant from this area was able to attend a focus group in Central. Healthcare professionals delivering the intervention in the West were unable to attend focus groups, but one acute physiotherapist and three technical instructors participated in individual telephone interviews. Four themes emerged:

Theme 1 - Variation of usual care and its impact on delivering the intervention

The frequency and format of usual community rehabilitation varied because of tailoring to individual need, the availability of resources and the provision of support services such as re-ablement and falls prevention classes. One carer described this variation as a "*postcode lottery*" (male carer, control group). In the control group, the initial contact with therapists often needed to be initiated by the patient, relying on their self-motivation, which was not necessary for the intervention group.

According to therapists, there was large geographical variation in usual care ranging from multiple same day appointments to no rehabilitation whatsoever. This variation affected how the enhanced rehabilitation intervention was delivered. One therapist commented that when she delivered the intervention to patients with minimal usual care she would "*spread out the sessions, and then just pushed [the patient] harder, in the two weeks*" (physiotherapist). In contrast, where a comprehensive rehabilitation programme was available, she would deliver intervention sessions weekly in the confidence that at the end

For peer review only

of the intervention period this provision would be continued through community run falls prevention or exercise schemes.

Therapists were concerned about supporting patients to set individualised goals when these might conflict with goals supported by other rehabilitation providers.

*"It was much harder when they had [another ongoing service], the re-ablement ones were much harder to actually, because somebody else was already setting what they were going to achieve" (*physiotherapist).

Theme 2 – The importance of goal-setting

Goal-setting was identified by therapists as playing an important role in engaging patients with their own recovery and in providing motivation to regain function and independence.

"I think [patients] probably more motivated because they can see the steps, to getting to that point. And why you are doing it" (physiotherapist).

The patient-held goal setting diary were appreciated by participants, as it gave them a direct focus and accountability for their goals.

*"You feel as if you have got a goal to get to, because you have put it in that book and you have got a goal." (*female patient, intervention group).

Therapists felt that the workbook and diary enabled patients to be more involved in their rehabilitation.

Theme 3 - The role of the therapist in providing reassurance about safe physical activities

For peer review only

The majority of patient participants reported anger and frustration when their physical ability to progress did not match their expectations, and they remained dependent on others.

"*Being incapacitated infuriated me so much"* (female patient, intervention group).

It was at this point that the physiotherapist played a pivotal role in managing expectations and reassuring patients that they were progressing normally. In the absence of this support there was a risk of patients losing motivation. This reassurance was important for giving patients the confidence to perform physical activities, as there was an underlying concern that they may otherwise do exercises which may be harmful.

"I had the security to know they were the right exercises, somebody there who gave them to me and you know they are qualified and they are telling you the right thing to do" (female patient, control group).

For participants in the control group, this lack of reassurance was a particular problem. Patients received a list of activities to avoid (hip precautions), but some were given no information about what exercises and activities were safe to perform, and wanted access to; "*somebody I could have just picked up the phone and said, how about this, should this be happening*" (female patient, control group).

Both groups identified this initial contact with therapists as vital for building their confidence and supporting their self-motivation for recovery.

"Once you have the information and the guidance on what to do, what not to do, I think we are intelligent enough to go away and do it, but it is just that initial guidance... we might be capable but you still need guidance" (male patient, control group).

For peer review only

Patients emphasised the importance of the intervention sessions in allowing them time to discuss their individual problems, particularly in the early stages of rehabilitation. This was facilitated by their relationship with the therapist or therapy team, where they felt comfortable enough to ask questions without fear of being dismissed or considered a nuisance. This was in contrast with how they felt in the acute hospital, or in usual care, where they were less informed about the processes and unfamiliar with the staff. A good relationship with their therapist underpinned successful rehabilitation, and enabled them to engage in, and take responsibility for, their role within the recovery process.

"I felt as though it was a sort of team effort, and she [the therapist] was sort of team leader, and knew what to do, and then it is sort of from part of the team if you like." (male patient, intervention group).

Theme 4 - Acceptability of the extra therapy sessions

Patient feedback on the intervention workbook varied. Some patients appreciated the explanation of the mechanics of their fracture and their better understanding of the surgery used to fix it.

"I thought it was good because it did explain things, it did explain to you what happens with a fracture" (female patient, intervention group).

Other patients reflected on the comfort that this additional information gave them.

"I didn't know what to expect but I found I read [the workbook] profusely every day, and I did, I found it very, very helpful. It made me feel that I wasn't on my own" (female patient, intervention group).

For peer review only

Other participants found the workbooks less useful.

"I sort of read it once and thought well you know this isn't very useful" (male patient, intervention group).

Without exception, the most useful aspect of the intervention was the extra time that participants received with the therapy teams. The goal-setting diary and information workbook were seen as useful supporting documents to these extra sessions. Whilst therapists acknowledged the complex nature of delivering intervention sessions in an environment of varied usual care, it was generally accepted that the extra sessions were a great benefit to patients. The analysis of the focus groups also led to the development of the GUIDE tool (Figure 1) which summarises the role of the therapist in the rehabilitation process, incorporating important factors identified by patients and their carers.

Unintended consequences

There were nine adverse events, six were serious, two resulted in re-admission to the acute hospital and there was one death; none were related to participation in the study.

Testing the intervention theory

Correlations between the Barthel Index and the three process measures of self-efficacy were statistically significant and suggested that higher levels of activities of daily living were associated with higher scores of self-efficacy (Appendix 6). Similarly higher scores in the FES-I represent a greater fear of falling, which was associated with lower levels of activities of daily living. The strongest correlation was with the FES-I.

For peer review only

Discussion

Summary of main findings

This study took place in three sites across North Wales. Recruitment to the study was more difficult in the West because of its rurality and also staff shortages. The recruitment rate was highest in Central; the retention rate was highest in the East. Usual rehabilitation care was very variable with a median of three appointments; the enhanced rehabilitation group received a mean of five additional therapy sessions. Variation in usual care affected how the enhanced rehabilitation intervention was delivered. TIs carried out most of the sessions, which consisted of practising exercises and activities of daily living, goal-setting, answering questions raised by the workbook and giving feedback on progress. Goal-setting had an important role in engaging patients in their own recovery, which was assisted by the workbook and diary. All of the returned goal-setting diaries had inputs from the therapy team, and half had written comments by the patients and carers. Some participants did not find the workbook and diary useful, but all valued the extra therapy sessions. The physiotherapist was very important for managing patients' expectations and for reassuring them about what physical activity was safe to perform. The lack of reassurance was particularly problematic for some in the control group. There were statistically significant correlations between three process measures of self-efficacy and the Barthel index, which supported the theory underlying the intervention.

Strengths and weaknesses

For peer review only

This was a mixed-methods process evaluation performed concurrently with a randomised feasibility study which has examined recruitment, reach, dose delivered, intervention fidelity, unintended consequences, contextual factors and underlying theory. Rates of recruitment and retention were low, and this process evaluation informs trial methods, as well as how to deliver the intervention for a future, definitive, phase III RCT. Although many commented that goal-setting was enhanced by the workbook and self-monitoring diaries, half of the collected workbooks were not completed, and some found them unhelpful. Feedback from participants and intervention delivery staff will result in further refinement of the workbook and diary and also the development of training materials before the definitive trial. Because this process evaluation was only part of a feasibility study it is not possible to comment on longer-term implementation of the enhanced rehabilitation intervention. The process data were analysed concurrently with the outcome data from the feasibility study, so the analysis of quantitative data was performed blind to treatment allocation; however, the qualitative findings and the feasibility study outcomes were discussed in relation to one another. It was not possible to collect data on usual rehabilitation from all participants in the intervention group, because the Therapy Manager software was only used by the rehabilitation teams in the Central area.

The participants did not include people with severe cognitive impairment, as the lack of mental capacity to give informed consent was an exclusion criterion. However, people with milder cognitive impairment, but who still had mental capacity, were not excluded.

The enhanced rehabilitation intervention was delivered by physiotherapists and Tis, with very little input from OTs. This was due to the availability of physiotherapists and the shortage of occupational therapists in this health board during the study period. We believe

For peer review only

that there is sufficient overlap in rehabilitation practice for these findings to be relevant to OTs as well. Also, the technical instructors who delivered most of the extra rehabilitation sessions worked with both physiotherapists and occupational therapists. The extra rehabilitation sessions concentrated on improving self-efficacy and personal goal-setting more than the practice of exercise and ADLs. We did manage to capture the practice of exercise and ADLs in participants' own time in a small number of participants who returned their diaries, however we do not know how often participants in the control group practised their exercises and ADLs outside of therapy sessions.

Despite being a feasibility study with a small sample size, it was possible to gather some evidence supporting the underlying theory with regard to the importance of self-efficacy.

Comparison with previous literature

Qualitative interviews of participants in the Exercise-Plus RCT in the United States, of a motivational intervention designed to increase adherence to rehabilitation exercise also found that identifying goals and improving self-efficacy were important, and an exercise booklet provided useful visual cues [25]. A good relationship with the therapist providing individualised care and verbal encouragement resulted in participants reciprocating their therapists' kindness by completing the exercise programme. They also described constraints to exercise such as unpleasant sensations of pain and fatigue, lack of time and space, and discontinuing their exercise once baseline function was restored. A qualitative study of a rehabilitation programme in Taiwan found that when therapists emphasised social support and resilience, patients developed more self-confidence and independence [26]. A process evaluation of a rehabilitation intervention in Sweden found that hip fracture had long lasting 'social and existential' effects on patients necessitating both physical and emotional support

For peer review only

during recovery [27]. The recent 'Hip Sprint'audit reviewed physiotherapy rehabilitation for hip fracture patients throughout the UK [28]. It found that usual rehabilitation care was variable with wide variation in the delay before home rehabilitation started, the amount and frequency of visits and the type of staff involved. Physiotherapists, and in particular physiotherapy assistants, provided most of the care.

Implications for a future definitive RCT

Several lessons have been learnt for delivering the enhanced rehabilitation intervention to elderly hip fracture patients with mental capacity in a definitive phase III RCT (Box 1). Recruitment was harder in rural areas, especially in areas with staff shortages, which will be an important consideration when choosing sites for the definitive trial. Research staff need to remain flexible, be prepared to recruit after discharge home, keep visiting potential participants and possibly delay recruitment until later after surgery. Employing therapists directly by the research team or secondment from the research delivery workforce would avoid them being pulled back to clinical work during staffing shortages. The workbook and goal-setting diary need to be refined further in the light of feedback from patients, carers and clinicians. A mnemonic (GUIDE) for therapists has been developed following the qualitative research (Figure 1), which will be useful as a training tool for the therapy teams prior to a definitive phase III RCT. The collection of usual rehabilitation care data could be enhanced by using a patient-held treatment log completed by visiting therapists.

Acknowledgments

For peer review only

The FEMuR team would like to thank all participants who took part in the study. The authors would also like to thank Health and Care Research Wales for supporting participant recruitment, and the healthcare staff at BCUHB who identified potential participants and delivered the intervention.

Contributors

NHW was the chief investigator and grant holder, was responsible for study design, conduct and analysis and had overall responsibility for the study and acts as guarantor. JLR was the study manager overseeing day-to-day conduct, participant recruitment, methodological input, conducted qualitative analysis for the focus groups. NUD was involved in participant recruitment, acquisition of quantitative and qualitative data and analysis. NT conducted the statistical analysis for the feasibility study. Her institution affiliation changed from Bangor University to the University of Sheffield during the study. VM was a co-investigator responsible for study design, provided health psychology expertise and methodological oversight throughout the study. MW was involved in the conduct of the study including maintenance of study documentation, acquisition of data, provided administrative support. AWP contributed to qualitative analysis of the focus groups. All authors were involved in drafting, revising and approving this manuscript.

Competing Interests

NHW, JLR, NUD, MW, NT, VM report a grant from NIHR HTA programme, for the conduct of the study. NHW reports additional grants from Public Health Wales, NIHR HTA and BCUHB, outside the submitted work.

Funding statement

For peer review only

This work was supported by the National Institute for Health Research's Health Technology Assessment Programme, grant number 11/33/03. The views and opinions expressed therein are those of the authors and do not necessarily reflect those of the HTA, NIHR, NHS or the Department of Health.

Ethics approval

The study received ethical approval from the UK NHS North Wales West Research Ethics Committee—West. Ref 13/WA/0402 and NHS Research and Development approval from the Betsi Cadwalader University Health Board (BCUHB) Internal Review Panel.

Availability of data and materials

The datasets used during the current study are available from the corresponding author on reasonable request.

References

1. Johansen A, et al., National Hip Fracture Database. National report 2013, C.E.a.E. Unit, Editor 013, Royal College of Physicians: London.

2. Bertram, M., et al., *Review of the long-term disability associated with hip fractures.* Injury Prevention, 2011. **17**(6): p. 365-370.

3. National Clinical Guideline Centre. Hip fracture: the management of hip fracture in adults| Guidance and guidelines | NICE. National Institute of Health and Care Excellence 2011. Accessed 2017. Available at http://www.nice.org.uk/guidance/cg124.

4. Handoll, H.H., et al., *Multidisciplinary rehabilitation for older people with hip fractures.*

For peer review only

Cochrane Database of Systematic Reviews, 2009(4).

5. Handoll, H.H., C. Sherrington, and J.C. Mak, *Interventions for improving mobility after hip fracture surgery in adults.* Cochrane Database of Systematic Reviews, 2011(3).

6. Crotty, M., et al., *Rehabilitation interventions for improving physical and psychosocial functioning after hip fracture in older people.* Cochrane Database of Systematic Reviews, 2010(1).

7. Williams NH, Roberts JL, Din NU, Charles JM, Totton N, Williams M, Mawdesley K, Hawkes CA, Morrison V, Lemmey A, Edwards RhT, Hoare Z, Pritchard AW, Woods RT , Alexander S, Sackley C, Logan P, Wilkinson C, Rycroft-Malone J. A multidisciplinary rehabilitation package following hip fracture: Fracture in the Elderly Multidisciplinary Rehabilitation (FEMuR). Health Technol Assess 2017; 21 (44).

8. Medical Research Council. Developing and evaluating complex interventions: new guidance. London: Medical Research Council, 2008.

9. Roberts JL, Din NU, Williams M, Hawkes CA, Charles JM, Hoare Z, Morrison V, Alexander S, Lemmey A, Sackley C, Logan P, Wilkinson C, Rycroft-Malone J, Williams NH. Development of an evidence-based complex intervention for community rehabilitation of hip fracture patients using realist review, survey and focus group. BMJ Open 2017; 7: e014362.

10. Williams NH, Hawkes C, Din NU, Roberts J, Charles JM, Morrison V, Hoare Z, Edwards RhT, Andrew G, Alexander S, Lemmey A, Woods B, Sackley C, Logan P, Hunnisett D, Mawdesley K, Wilkinson C. Fracture in the Elderly Multidisciplinary Rehabilitation (FEMuR): study protocol for a Phase II randomised feasibility study of a multidisciplinary rehabilitation package following hip fracture [ISRCTN22464643] Pilot feasibility Studies 2015; 1: 13.

For peer review only

11. Williams NH, Roberts JL, Din NU, Totton N, Charles JM, Hawkes CA, Morrison V, Hoare Z, Williams M, Pritchard AW, Mawdesley K, Hawkes CA, Morrison V, Lemmey A, Edwards RhT, Hoare Z, Pritchard AW, Alexander S, Lemmey A, Woods RT, Sackley C, Logan P, Edwards RhT, Wilkinson C. Fracture in the Elderly Multidisciplinary Rehabilitation (FEMuR): a phase II randomised feasibility study of a multidisciplinary rehabilitation package following hip fracture. BMJ Open 2016; 6: e012422.

12. Hodkinson HM. Evaluation of a mental test score for assessment of mental impairment in the elderly. Age Ageing 1972; 1: 233–238.

13. Steckler A, Linnan L (Eds.) Process evaluation for public health interventions and research. San Francisco: Jossey-Bass, 2002.

14. Moore G, Audrey S, Barker M, Bond L, Bonell C, Hardeman W, Moore L, O'Cathain A, Tinati T, Wight D, Baird J. Process evaluation of complex interventions: UK Medical Research Council (MRC) Guidance. MRC Population Health Science Research Network, 2014.

15. Mastersson-Allgar P. Advancing process evaluation research within the field of Neurological rehabilitation. PhD thesis, Bangor University, 2016.

16. Grant A, Treweek S, Dreischulte T, Foy R, Guthrie B. Process evaluations for cluster randomised trials of complex interventions: a proposed framework for designing and reporting. Trials 2013; 14: 15.

17. Oakley A, Strange V, Bonell C, Allen E, Stephenson J, RIPPLE Study Team. Process evaluation in randomised controlled trials of complex interventions. BMJ 2006; 332: 413-416.

18. Moore GF, Raisanen L, Moore L, Din NU,Murphy S. Mixed-method process evaluation of the Welsh National Exercise Referral Scheme. Health Education 2013; 113(6): 476-501.

For peer review only

19. Pawson R, Tilley N. Realistic evaluation, London: Sage, 1997.

20. Mahoney F, Barthel D. Functional evaluation: the Barthel index. Maryland State Med J. 1965;14:56–61.

21. Schwarzer R, Jerusalem M. Generalized self-efficacy scale. In: Weinman J, Wright S, Johnston M, editors. Measures in health psychology: a user's portfolio causal and control beliefs. Windsor, UK: NFER-NELSON; 1995. p. 35–7.

22. Hauer K, Yardley L, Beyer N, Kempen G, Dias N, Campbell M, et al. Validation of the Falls Efficacy Scale and Falls Efficacy Scale International in geriatric patients with and without cognitive impairment: results of self-report and interview-based questionnaires. Gerontology. 2010;56:190–9.

23. Yardley L, Beyer N, Hauer K, Kempen G, Piot-Ziegler C, Todd C. Development and initial validation of the Falls Efficacy Scale-International (FES-I). Age Ageing. 2005;34:614–9.

24. Resnick B, Jenkins LS. Testing the reliability and validity of the self-efficacy for exercise scale. Nurs Res. 2000;49:154–9.

25. Resnick B, Orwig D, Wehren L, Zimmerman S, Simpson M, Magaziner J. The Exercise Plus program for older women post hip fracture: participant perspectives. Gerontologist. 2005;45:539–44.

26. Huang T, Acton G. Ways to maintain independence among Taiwanese elderly adults with hip fractures: A qualitative Study**.** Geriatric Nurs. 2009;30:28-35

27. Ziden L, Wenestam C, Hansson-Scherman M. A life-breaking event: early experiences of the consequences of a hip fracture for elderly people. Clin Rehab. 2008;22:801–11**.**

28. Royal College of Physicians. Recovering after a hip fracture: helping people understand physiotherapy in the NHS. Physiotherapy 'Hip Sprint' audit report 2017. London: RCP, 2018.

For peer review only

Table 1 Process evaluation questions and methods for evaluating

Component	Process evaluation questions	Research methods	Stage of study to collect data
Recruitment of sites and rehabilitation teams	How are sites and teams recruited?	Documentation of recruitment process by research team	Pre-intervention
	Which sites and teams agree to participate?	Quantitative comparison of recruited and non-recruited sites	
Response of rehabilitation teams	How is the enhanced intervention adopted by the rehabilitation teams	Quantitative examination of case report forms and qualitative interviews of rehabilitation team members	During and following the intervention
Recruitment and reach in patient and carer participants	How many are recruited into the feasibility study? Are they representative?	Quantitative comparison between feasibility study and anonymised cohort	During the intervention
	Who is recruited into the feasibility study? What are the reasons for non-recruitment?	Examination of recruitment log	During the intervention
Intervention delivery	What rehabilitation intervention is delivered? Is it what was intended by the researchers?	Quantitative examination of case report forms and of electronic data entered onto Therapy Manager software	During the intervention
Delivery to individuals	What intervention is delivered to each participant?	Quantitative examination of case report forms and of electronic data entered onto Therapy Manager software	During the intervention
	Is the delivered intervention the one intended by the researchers?	Measurement of intervention fidelity: completion of workbook tasks, completion of diaries, number and content of therapy sessions	During the intervention
Response of individual patients to the enhanced intervention or usual rehabilitation	How do the patient participants respond?	Qualitative analysis of focus group data about patient participants' experience and response to the intervention and to usual care	Following the intervention
Response of carer participants	Effects on carers	Qualitative analysis of focus group data about carers' experiences	Following the intervention
Unintended consequences	Are there unintended changes in processes and outcomes related to the intervention and unrelated to care?	Quantitative examination of adverse effects reports, health service activity data from patient completed questionnaires and routinely collected electronic sources. Qualitative analysis of focus group data from patients and	During and following the intervention

For peer review only

		their carers	
Theory	What theory has been used to develop the intervention?	Quantitative data analysis of process outcome measures to assess predicted relationships	Following the intervention
Context	What is the wider context in which the feasibility study is conducted?	Realist review of the rehabilitation literature, survey of current rehabilitation practice, focus groups of patients, carers and rehabilitation professionals. Quantitative comparison with anonymised cohort	Pre-intervention (phase I study) and during the intervention

For peer review only

Table 2 Eligibility, recruitment and retention rates according to acute hospital site

Number of Patients	West	Central	East	Total
**Screened (rate)**	147	235	211	593
**Eligible (rate)**	75 (51%)	103 (44%)	88 (42%)	266 (45%)
**Recruited (rate)**	11 (15%)	35 (34%)	16 (18%)	62 (23%)
**Retained (rate)**	4 (36%)	29 (83%)	16 (100%)	49 (79%)

For peer review only

Table 3 Focus group participants' characteristics

Participant type	Location	Attendees
Patient and carers in control group	East	2 female patients, 1 male patient, 2 male carers (n=5)
Patient and carers in control group	Central	2 female patients, 1 male patient, 1 female carer (n=4)
Patient and carers in intervention group	East	3 female patients (n=3)
Patient and carers in intervention group	Central	2 male patients, 2 female patients, 1 male carer, 2 female carers (n=7)
Healthcare professionals	East	Clinical specialist physiotherapist, 2 orthopaedic physiotherapists, physiotherapy technical instructor (n=4)
Healthcare professionals	Central	Orthopaedic acute physiotherapist, rotational physiotherapist, physiotherapy technical instructor (n=3)

For peer review only

Box 1 Ten lessons learnt for a future definitive randomised controlled trial

Sites	1	Consider staff availability and rurality when recruiting sites
Therapy staff	2	Consider employing therapy staff directly or second from research delivery teams
Participant recruitment	3	Recruitment flexibility – including after discharge home
	4	Keep visiting potential participants
	5	Delay recruitment until later after surgery
Trial-specific training	6	Use the mnemonic GUIDE as a training aid
	7	Stress the importance of the first home visit to reassure participants about safe activities
	8	Value the emotional support provided by the technical instructors
Usual care recording	9	Patient-held treatment log completed by visiting therapists.
Workbook	10	Further refine the workbook in the light of feedback

For peer review only

Figure Legends

Figure 1 GUIDE mnemonic for therapists involved in rehabilitation following hip fracture

For peer review only

For peer review only

For peer review only

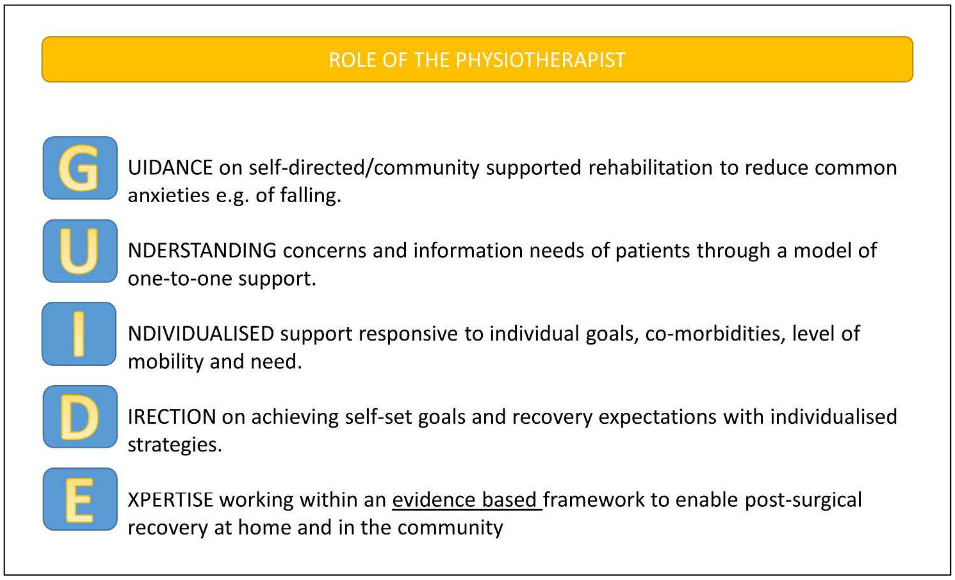

Figure 1. GUIDE mnemonic for therapists involved in rehabilitation following hip fracture

275x190mm (300 x 300 DPI)

Appendix 1 Case report form

Session number:

Patient Study ID: ☐☐☐☐☐

Date: ____ /____ /________ **Total amount of time used for this session:**

Role(s) of person/people conducting visit: Occupational therapist / Physiotherapist / OT assistant / Physiotherapy assistant / Technical instructor / Other

Location of session: Patient's own home / Care home where patient lives permanently / Community hospital outpatients / Acute hospital outpatients / ________________________ Other

1. Please indicate what ***indirect*** care activities you carried out for the patient during this session, and for how long:

Activity	Time (e.g. 10 minutes)	Further details
Referring to another service		
Phone contact with patient		
Contact with MDT about patient		
Patient admin		
Travel to and from session		
Other, please specify.		

2. Please indicate what ***direct*** care activities you carried out for the patient during this session, and for how long:

Activity	Time (e.g. 20 minutes)	Further details
Assessment/re-assessment		
Physical exercises		
Activities of daily living practice		
Workbook		
Discussion of care and progress		

For peer review only

Activity	Time (e.g. 20 minutes)	Further details
Discussion of referral to a follow on service e.g. social groups, falls groups, outpatients physio		
Travel to and from session		
Other, please specify		

3. If you spent time on the ***workbook*** in the session, please indicate what this involved:

Activity	Time (e.g. 5 minutes)	Further details
Goal setting		
Reviewing progress		
Answering patient questions stimulated by workbook		
Emotional support		
Other, please specify		

For peer review only

Appendix 2 Focus group topic guides

Patient and carer topic guide – control group

- What was your experience of the rehabilitation you received after your hip fracture, specifically regarding physiotherapy and exercise?
- What went well, what could be improved? Having experienced fracture and the rehabilitation involved, what would you recommend to improve care?
- If there are any attending the focus group, what were the experiences of participants who were discharged to nursing/residential care, or those who were more dependent on carers?

- Did your therapists give you exercises to do?
    - How confident did you feel about practising them?
    - Did your confidence change over time? What changed this?
- Were you given any written information to keep with you?
    - How helpful was this? Was there any other information you would have found useful?
- How involved did you feel in planning your recovery and rehabilitation?
- Often people say they were afraid of falling again after having a hip fracture, did you experience this?
    - Was there anything in your rehabilitation that helped reduce the fear of falling?
    - Did you attend a falls prevention class? How did you find out about this and was it useful?
- How did you feel about being approached to take part in the study?
    - Did you understand the information you were given?

For peer review only

  - What made you decide to take part?
  - How did you feel about the questions you were asked in the hospital and at the follow up, especially about how long they took and how well you were able to understand the questions and their relevance?
- How did you feel about not knowing which study group you would be in when you agreed to take part?
  - How would you have felt if you had been in the other group?
- Did you do some exercise tests with your community physiotherapists at the end of the study?
  - How did you feel about being asked to do these?
- Has anything changed for you as a consequence of being in the study?
  - If you were asked to take part now, would you make the same decision?

Patient and carer topic guide – intervention group

- What was your experience of the rehabilitation you received after your hip fracture?
  - What went well, what could be improved?
  - If there are any attending the focus group, what were the experiences of participants who were discharged to nursing/residential care, or those who were more dependent on carers?
- TASK - Rank the following aspects of the intervention according to how useful they were:
  - Extra time with a therapist
  - Information in the workbook
  - Being involved in setting your own goals

For peer review only

    - Keeping a diary to look at progress
- Discussion of ranking
- Did you feel confident to suggest goals to your therapists and how did that help you?
- What did you like best about the workbook?
    - Was there anything you felt the workbook was missing?
- Were you aware of the extra sessions which you received because you were in the study?
    - Were these sessions used differently to the care as usual sessions?
    - Did you notice any improvements in your recovery as a result of this extra time?
- Did your therapists give you exercises to do?
    - How confident did you feel about practising them?
    - Did your confidence change over time? What changed this?
- Often people say they were afraid of falling again after having a hip fracture, did you experience this?
    - Was there anything in your rehabilitation that helped reduce the fear of falling?
    - Did you attend a falls prevention class? How did you find out about this and was it useful?
- How did you feel about being approached to take part in the study?
    - Did you understand the information you were given?
    - What made you decide to take part?
    - How did you feel about the questions you were asked in the hospital and at the follow up, especially about how long they took and how well you were able to understand the questions and their relevance?

For peer review only

- Did you do some exercise tests with your community physiotherapists at the end of the study?
    - How did you feel about being asked to do these?
- How did you feel about not knowing which study group you would be in when you agreed to take part?
    - How would you have felt if you had been in the other group?
- Has anything changed for you as a consequence of being in the study?
    - If you were asked to take part now, would you make the same decision?

Healthcare professional topic guide

- Experiences of the enhanced rehabilitation intervention
    - What went well?
    - What could be improved?
- Experiences of extra therapist time
- Awareness of extra time available to patients in the study
    - How did if work, what went well what needs to be improved
    - How did you use the extra time with patients? Do you think it made a difference?
- Views of the Workbook
    - Did you use it?
    - If so how, what was useful, what could be improved, was anything missing you think would have been helpful?

For peer review only

- Do you feel the intervention made any difference to the way patients engaged with you and participated in their rehabilitation?
  - If so how/if not why?
  - What was your experience the goal setting and feedback in the work book? Similar/different to usual way you work/good points/areas for improvement
  - What was your experience of the information sections? Useful to you/useful to patients/anything you weren't aware of before/ anything that was missing?
- Experiences of working with patients with cognitive impairments and their carers
  - How did the intervention go?
- Experiences of working with patients who were discharged to long term residential/nursing care
  - How did the intervention go?
- Feasibility of physiotherapists conducting the physical measures at 3 month follow-up
- Acceptability of the outcome measures for patients
  - Timing; Number; Content
- Anything else we haven't talked about you think has been important in working with this intervention?

For peer review only

Appendix 3 Rehabilitation pathways in usual care on discharge from the central acute hospital following surgical repair of hip fracture

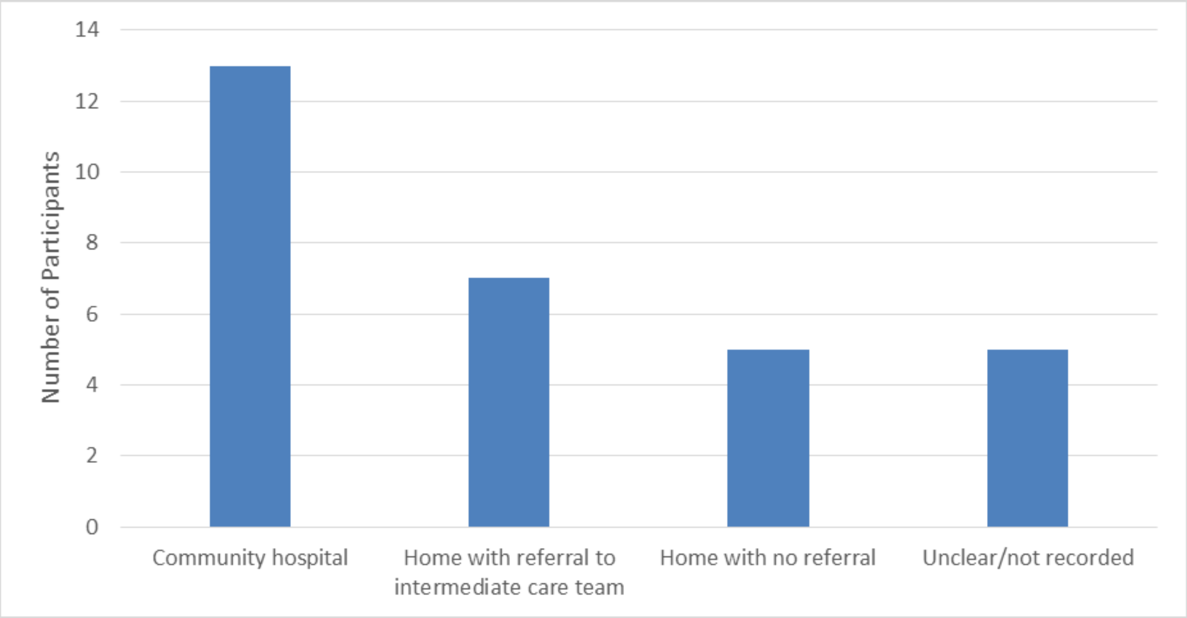

For peer review only

Appendix 4 Percentage of home visits conducted by different members of the rehabilitation team for usual care

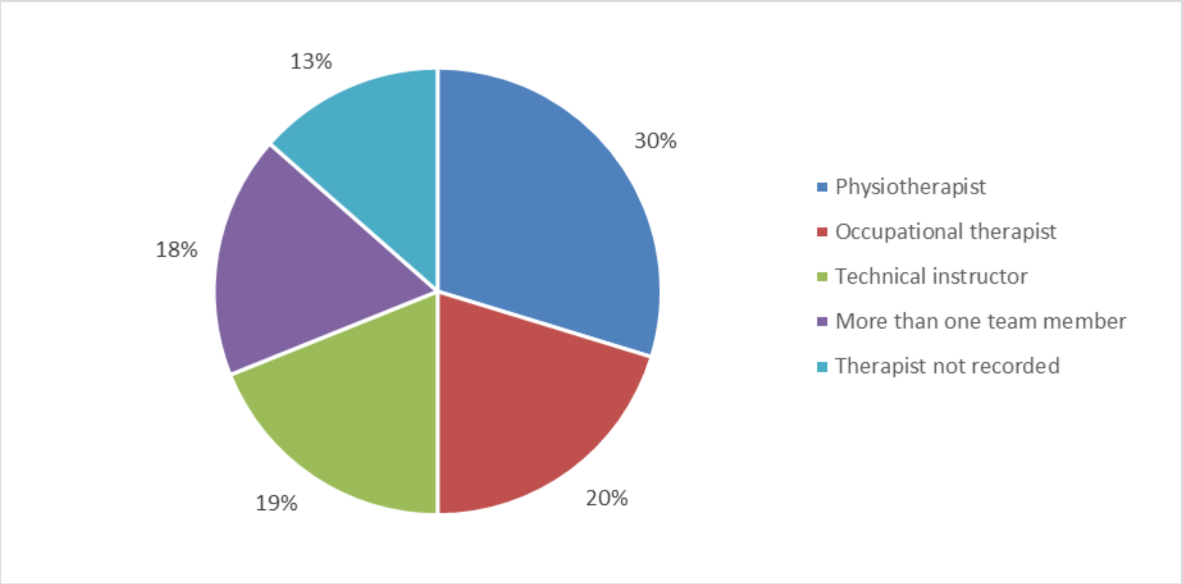

Appendix 5 Reasons for missing data on intervention session use

Reason	No. of participants	Area
Withdrawn	4	Gwynedd, Anglesey, Conwy and Denbighshire
Area not covered by therapist	1	Gwynedd and Anglesey
Declined intervention sessions	1	Conwy and Denbighshire
Could not be contacted to arrange sessions	1	Conwy and Denbighshire
Received intervention but therapist not returned paperwork	1	Gwynedd and Anglesey
Therapist delivering sessions moved to different area and could not complete intervention	1	Gwynedd and Anglesey

For peer review only

For peer review only

Appendix 6 Correlation analysis of Barthel Index with measures of self-efficacy

Self-Efficacy Measures	Barthel Index at baseline<br>Correlation (95% Confidence Intervals)	Barthel Index at 3 months<br>Correlation (95% Confidence Intervals)
General Self-Efficacy Scale (Baseline and 3 Months)	r_{55}= 0.43 (0.12 to 0.68), p=0.001	r_{37}= 0.59 (0.23 to 0.82), p<0.001
Falls Efficacy Scale – International* (3 Months)	Not Collected	r_{34}= -0.68 (-0.83 to -0.45), p<0.001
Self-Efficacy for Exercise Scale (3 Months)	Not Collected	r_{33}= 0.62 (0.26 to 0.82), P<0.001

* High values represent high levels of fear of falling

For peer review only
